# Cascaded Broadband Low-Frequency Microwave Absorption Covering P- to C-Band in Ultra-Thin Metamaterials via Synergistic Local‑Field and Loss‑Field Enhancement

**DOI:** 10.1007/s40820-026-02245-1

**Published:** 2026-06-22

**Authors:** Qian Yang, Hongbo Hou, Yongxi Lu, Zhongqiu Guo, Jiaxu Sun, Peng Zhang, Tian Yang, Fanbin Meng

**Affiliations:** Key Laboratory of Advanced Technologies of Materials (Ministry of Education), School of Materials Science and Engineering, Southwest Jiaotong University, Chengdu, 610031 People’s Republic of China

**Keywords:** Low-frequency, Local field enhancement, Metasurface, Mechanical flexibility

## Abstract

Low-frequency radar waves, particularly in the P-band, present a significant stealth challenge due to the inherent trade‑offs among strong absorption, broad bandwidth, and ultra-thin thickness. These limitations arise from the conflict between structural thickness and wavelength, impedance-matching difficulties, and weakened loss mechanisms. To overcome these constraints, a new strategy for synergistic enhancement of the local field and electromagnetic loss field of metamaterials has been proposed. By employing metasurface structures for local‑field enhancement, strong absorption is achieved at ultra-thin thicknesses. Furthermore, dielectric, magnetic, conduction, and structural resonance losses are integrated to enable strong, broadband absorption. Herein, a double-layer metasurface array is designed and integrated onto a polydimethylsiloxane/flake carbonyl iron high‑loss dielectric substrate. The resulting composite exhibits exceptional performance in the 1.77–2.85 GHz range at a thickness of only 3.78 mm (~ 0.022 λ), with an absorption rate exceeding 90%, and the absorption rate within the 1–6 GHz range can exceed 60%. It also demonstrates good mechanical flexibility and stability. The proposed local‑field enhancement principle provides a new route to bypass the quarter-wavelength limitation of traditional absorbers, while its ultra-thin, broadband, and flexible integrable features highlight its potential for efficient conformal integration on complex curved surfaces.

**Supplementary Information:**

The online version contains supplementary material available at 10.1007/s40820-026-02245-1.

## Highlights

- A “local field enhancement” strategy based on field-loss co-design, offering a new approach to overcome the performance trade-offs in low-frequency absorption.
- A subwavelength coupled metasurface array is used to replace the traditional reflective layer, breaking the thickness limitation imposed by the quarter-wavelength theory.
- Enabling vertical cascading of absorption performance, moving beyond the constraint of zero transmission in traditional absorbers and opening possibilities for multi-layer design.

**Supplementary Information:**

The online version contains supplementary material available at 10.1007/s40820-026-02245-1.

## Graphical abstract

**Figure:**
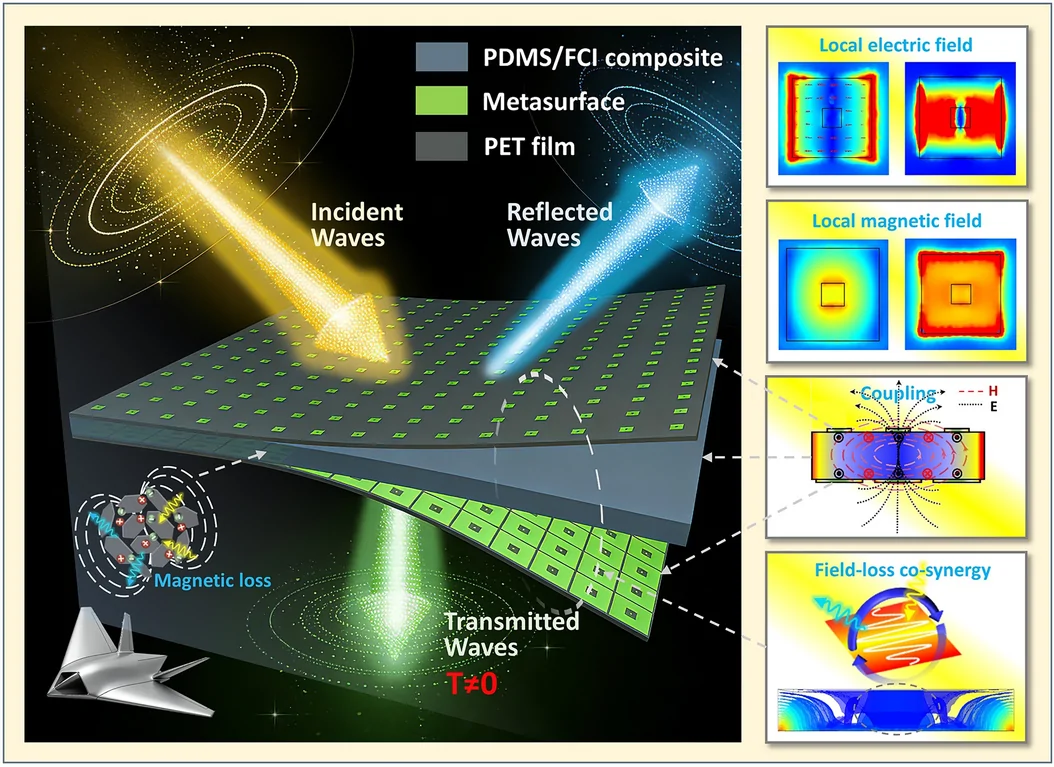

## Introduction

With the rapid advancement of electromagnetic (EM) interference and radar detection technology, the EM pollution affecting modern stealth aircraft has expanded from the conventional high-frequency domain to the low-frequency (LF) domain [1, 2]. This shift imposes critical demands on absorbing materials, which must simultaneously deliver strong LF absorption, broad bandwidth, and small thickness [3, 4]. In practical applications, LF absorbers must also exhibit mechanical flexibility to ensure intimate contact with complex‑geometry aircraft skins. Consequently, developing high‑performance, flexible absorbing materials suitable for the LF domain has become an urgent imperative for enhancing the survivability and EM compatibility of stealth platforms. Currently, the LF absorption performance has been significantly improved through compositional and microstructural optimization, yet most efforts remain concentrated on the S‑ and C‑bands, with limited progress given to the L‑ and P‑bands [5, 6]. This is mainly attributed to the excessively large theoretical thickness caused by the wavelength, the lack of effective loss mechanisms, and the difficulty in impedance matching [7, 8]. These issues collectively hinder broadband impedance matching, the concurrent optimization of constitutive parameters, and the attainment of sufficient magnetic/dielectric loss in the LF region. Therefore, in previous LF work, the effective absorption bandwidth could only cover 3.92–6.32 GHz at 4.4 mm [9]. To achieve effective absorption in the L-band, a matching thickness of up to several tens of millimeters was required [10].

Metasurfaces (MSs) are two-dimensional artificial structures composed of subwavelength unit cells arranged periodically, enabling flexible manipulation of the amplitude, phase, and polarization of EM waves [11–13]. This capability presents a promising alternative to overcome these limitations by shifting the design paradigm from intrinsic material properties to engineered artificial structures. However, current designs predominantly rely on single-mode electrical resonance, with insufficient exploration of multimode coupling such as magnetic and Fano resonances [14, 15]. Moreover, most MS absorbers utilize low-loss dielectric substrates, limiting energy dissipation to ohmic losses in metallic resonators and lacking synergy with high-loss absorbers [16, 17]. Recently, integrating MS structures with lossy substrates has emerged as a more effective strategy to improve impedance matching and broaden absorption bandwidth. Furthermore, through structural design, the material can be engineered into a three-dimensional (3D) metastructure, which improves EM wave absorption performance without modifying its intrinsic EM properties [18, 19]. Efficient P-band absorption at relatively small thicknesses has been reported by embedding a three-layer cross-shaped frequency-selective surface into a conventional NiZn ferrite absorber [20], or by combining the inherent magnetic losses of NiCuZnBi ferrite with the macrostructure design to realize absorption from 0.43 to 1.0 GHz at 127 mm [21]. Despite these advances, achieving efficient LF absorption within sub‑wavelength thickness remains challenging due to intrinsically weak dielectric loss, insufficient magnetic loss from permeability dispersion, and limited microstructural control over long‑wavelength waves. Moreover, although quarter‑wavelength interference designs can enhance absorption, they often result in impractical thicknesses and fail to support multilayer integration due to zero transmission (*T* = 0) when a metal reflector is used [22, 23].

In this work, a cascaded metasurface flexible absorber composite (CMFAC) was designed to overcome these limitations. The CMFAC integrates a flexible polydimethylsiloxane/flake carbonyl iron (PDMS/FCI) lossy substrate with LF-responsive MS unit arrays, thereby replacing the conventional metal reflector layer. By enabling controllable transmission (*T* ≠ 0), this design not only breaks the conventional thickness limit for resonant absorption in the LF band but also allows for multilayer cascading. Specifically, the untrapped EM waves can continue to propagate through the bottom MS and be further absorbed by subsequent layers. This cascadable characteristic provides new degrees of freedom for optimizing absorption performance. While maintaining a millimeter-scale ultra-thin thickness (3.78 mm), it can be tailored for specific frequency bands—such as the P, L, and S bands—to achieve broadband and efficient absorption. In contrast to conventional impedance‑matching designs that employ MS layers with high sheet resistance (*Rₛ*), this work utilizes low *Rₛ* materials to fabricate the MS layers. This configuration excites strong resonances, confining the incident electric fields in the vicinity of the MS patterns and inducing intense interactions with the underlying lossy substrate, thereby facilitating efficient energy transfer. When the frequency of the incident EM wave matches the resonant currents, it promotes highly concentrated localized EM fields in specific regions. Then, by utilizing the interaction between the localized fields and the inherent EM losses arising from polarization relaxation and hysteresis loss, we establish a field-loss co-design that significantly enhances energy dissipation. The fabricated lossy substrate exhibits exceptional mechanical resilience, characterized by negligible permanent deformation and a high recovery ratio under cyclic mechanical loading. This strategy facilitates broadband and deep attenuation in multilayer flexible absorber structures, thereby expanding their application potential in complex EM environments and on flexible platforms with stringent density and thickness requirements.

## Experimental Section

### Raw Materials

Raw materials used were: PEDOT:PSS dispersion (PHV500, with a solid content of 1.0–1.3 wt%) (Nanchang Tongsheng New Energy Science and Technology Co., Ltd.), highly absorbent polymer (SAP) beads (Amazon (QMays)), deionized water (Produced using a laboratory-scale purification system), dimethyl sulfoxide (DMSO), anhydrous ethanol, acetone, and glacial acetic acid (Chengdu Haihong Experimental Instrument Co., Ltd.), KH-560 silane coupling agent (Shanghai Maclin Biochemical Technology Co., Ltd.), FCIs (BASF SE), polyethylene terephthalate (PET) film (Suzhou Tengcan Adhesive Products Co., Ltd.), Ni-plated PET film (Suzhou Zhongjiate Electronic Technology Co., Ltd.), and PDMS (Dow Corning Co.). All chemicals were used as received without further purification.

### Preparation of the Metasurface Absorber

#### PEDOT:PSS Conductive Ink

The experimental procedure for the PEDOT:PSS conductive ink was carried out according to the method described in our previous work, with minor modifications. The *R*_*s*_, thickness control, uniformity, and environmental stability of the PEDOT:PSS film have been systematically characterized in our previous work [24]. Briefly, the solid content of the ink we prepared is 9%, and the volume ratio of deionized water to DMSO is 7:3. The formulated conductive ink exhibits moderate viscosity and favorable flowability for processing (Fig. S13).

#### KH-560 modified FCIs

Add 200 mL of anhydrous ethanol, 2.76 g of deionized water, and 12 g of KH-560 to the beaker and stir evenly. Add glacial acetic acid to adjust the pH value of the solution to 4.0–5.0, and ultrasonically disperse for 5 min. Weigh 200 g of FCIs and place it in a beaker. Stir mechanically for 2 h, then let the reactants stand for 6 h. Finally, wash them 3–5 times with anhydrous ethanol and dry them at 80 °C to obtain the M-FCIs. The specific preparation process is shown in Fig. S12a.

#### Preparation of the MS layer

The MS layer based on PEDOT:PSS conductive ink was applied via screen printing according to the method described in our previous work, with PET film serving as the substrate [24]. The *R*_*s*_ of the PEDOT:PSS film was controlled by adjusting the number of printed layers. Side-view SEM images at different layer counts are shown in Fig. S14. The MS layer based on Ni-plated PET film is fabricated via laser etching. The specific preparation detail is shown in Fig. S12b.

#### PDMS/FCI Composite

Initially, the M-FCIs were mechanically stirred with the PDMS base material at the specified mass ratio to ensure uniform dispersion of the M-FCIs within the PDMS matrix. The PDMS curing agent was then added to the mixture (weight ratio of 1:10 to the base material), and mechanically stirred for 5 min. Finally, the mixture was poured into different PTFE molds. The PTFE molds used for preparing the PDMS/FCI composites of different specifications are shown in Fig. S15, after 12 h of curing, and then de-molded to obtain the PDMS/FCI composites (Fig. S12c). The PDMS/FCI composites prepared are systematically designated as PDMS/FCI@x, where x represents the mass fraction of M-FCIs in the composites. Composites with M-FCIs mass fractions of 5, 25, 50, 75, and 90 were prepared. Their densities as a function of M-FCIs mass ratio are shown in Fig. S16.

#### CMFAC

The top and bottom layers are the prepared MS layers, with the intermediate layer being the PDMS/FCI@75 composite, layered from bottom to top. The PET film faces outward, while the MS layer faces inward. This structure effectively encapsulates the conductive pattern, protecting it from mechanical wear and environmental corrosion that could compromise its performance. Pressure was applied to the top layer, and the assembly was allowed to cure preliminarily at 65 °C for 24 h, then let it stand for at least 7 days to achieve full performance stabilization, and then obtain the CMFAC.

### Simulation Details

The S-parameters and far-field distributions were simulated using CST Microwave Studio. The excitation port and boundary conditions used in the simulation are shown in Fig. S17 and Table S1. The simulation frequency range was 0.3–6.0 GHz. The near-field electric and magnetic field distribution, surface current, and loss distribution characteristics were simulated using COMSOL Multiphysics. The solver, excitation source, boundary conditions, and grid division used in the simulation are shown in Table S4.

### Characterization and Instruments

The thickness was tested by a digital thickness gauge, which measured the thickness in at least 10 positions in different directions. Fourier-transform infrared (FT-IR) spectroscopy was performed using an infrared spectrometer. XPS was performed using an X-ray photoelectron spectrometer, all binding energies were calibrated by referencing the C 1* s* peak at 284.8 eV. TGA was conducted on a thermogravimetric analyzer from 30 to 900 °C at a constant heating rate of 10 °C min^−1^ under a nitrogen atmosphere. The density was tested by a density tester. The *R*_*s*_ of the MS pattern was measured via the four-probe method. The surface and cross section of the samples were observed using an SEM. The EDS was employed to analyze the elemental composition and distribution on its surface. A series of coaxial rings made of the PDMS/FCI composites was pressed using a special mold with an outer diameter of 7.00 mm and an inner diameter of 3.04 mm (Fig. S15c). Subsequently, the complex permittivity and permeability were measured based on the coaxial-line method using a VNA, Ceyear, 3671D vector network analyzer in the range of 0.3–18.0 GHz. The reflectivity of CMFACs was measured using the free-space method for vertically incident waves across the 1.0–18.0 GHz frequency range. The tensile and compression properties were evaluated using the INSTRON universal testing machine.

## Results and Discussion

### Microstructure and Mechanical Properties

**Fig. 1:**
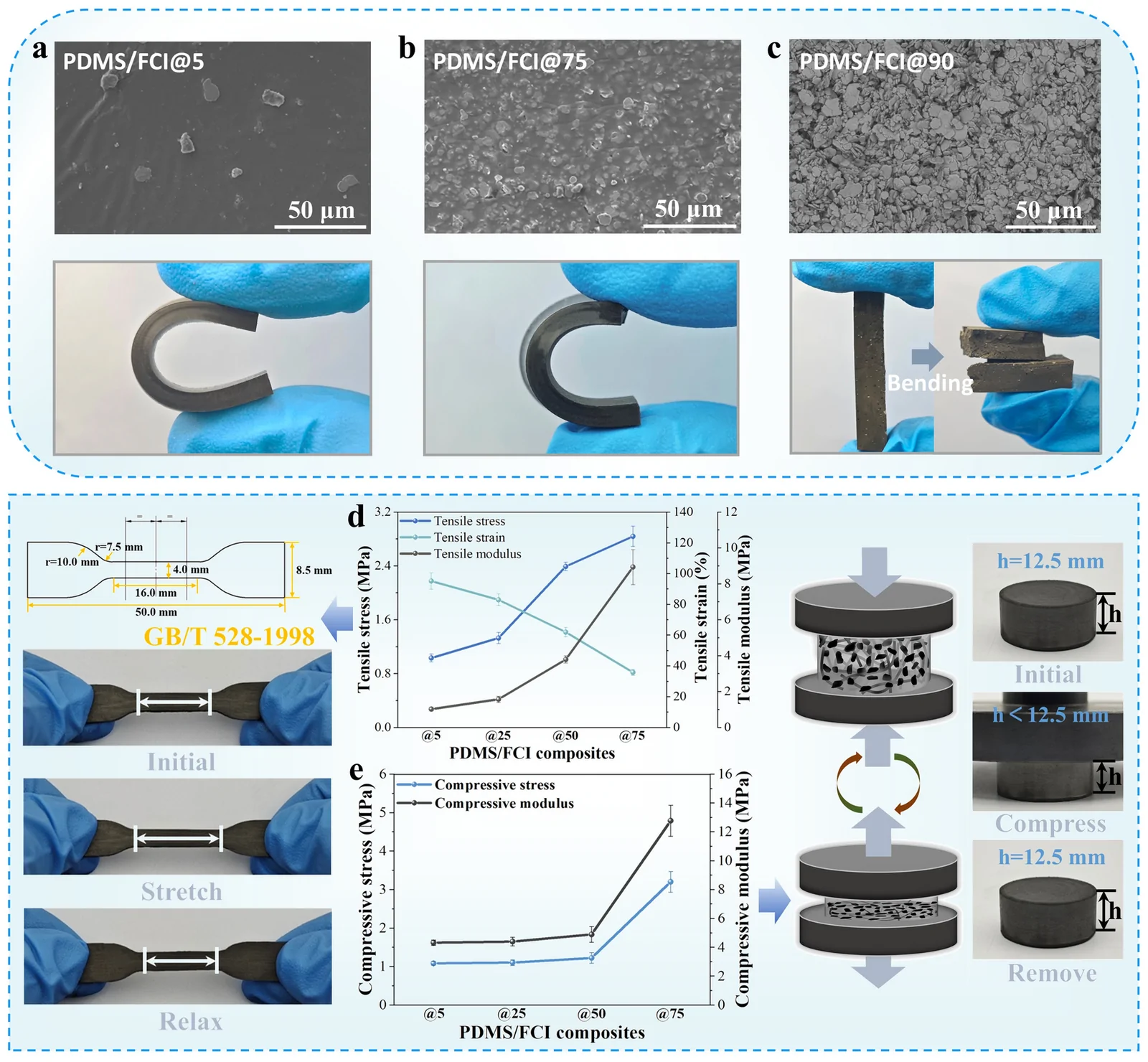
Surface SEM and macroscopic photographs of the bent PDMS/FCI composites: **a** PDMS/FCI@5; **b** PDMS/FCI@75; **c** PDMS/FCI@90; **d** Tensile properties, testing standards and the recovery process;** e** Compressive performance, cycle behavior, and the recovery process

To develop a flexible and efficient LF absorber, a mechanically flexible EM-loss substrate was fabricated using a PDMS composite filled with KH‑560 modified FCIs (M-FCIs). As shown in Figs. 1a–c and S1–S2, increasing the filler loading from 5 to 90% shifts the dispersion of M‑FCIs from uniform to severely agglomerated. At 90% loading, a continuous PDMS phase can hardly form, resulting in a complete loss of flexibility and a sharp decline in mechanical properties; the composite becomes brittle and fractures under slight force. The tensile stress–strain curves (Figs. 1d and S3) reveal a clear trade-off: higher M‑FCIs content increases tensile stress and modulus but reduces strain. Notably, even at 75% filler loading, the composite retains significant tensile recovery. Similarly, static compression tests at 25% strain (Figs. 1e and S4) show that both compressive stress and modulus rise with increasing M‑FCIs loading. Further, cyclic compression tests (10%–60% strain, Fig. S5) demonstrate that the 75% M‑FCIs composite exhibits minimal permanent deformation and high resilience. The excellent elasticity originates from the cross‑linked elastic PDMS network that efficiently stores and releases strain energy, while uniformly dispersed flaky particles reinforce structural integrity without severely compromising flexibility [25, 26]. This remarkable enhancement in overall mechanical properties is primarily attributed to the strong interfacial bonding between the M‑FCIs and the PDMS matrix. Specifically, the characteristic infrared peaks confirmed the presence of the KH-560 coating (Fig. S6a), which was further definitively verified by X-ray photoelectron spectroscopy (XPS) analysis demonstrating the successful chemical grafting onto the surfaces of the FCIs through the formation of robust interfacial covalent bonds (Fig. S6b). Consequently, thermogravimetric analysis (TGA) demonstrated that this dense silane coating acts as an effective physical barrier, significantly enhancing the oxidation resistance and thermal stability of the M-FCIs (Fig. S6c). Macroscopically, the tensile fracture surface morphology of the PDMS/FCI@75 composite (Fig. S2c) clearly reveals that the M-FCIs are tightly embedded within the PDMS matrix, with most fillers exhibiting fracture rather than pull-out. These multi-scale observations confirm that the KH-560 surface modification significantly improves interfacial adhesion, leading to enhanced tensile strength, modulus, and compressive performance at high filler loadings. Ultimately, this robust compressive recovery ensures structural and functional integrity under repeated stress, serving as a prerequisite for flexible wave‑absorbing applications.

### Electromagnetic Parameters and Absorption Performance

**Fig. 2:**
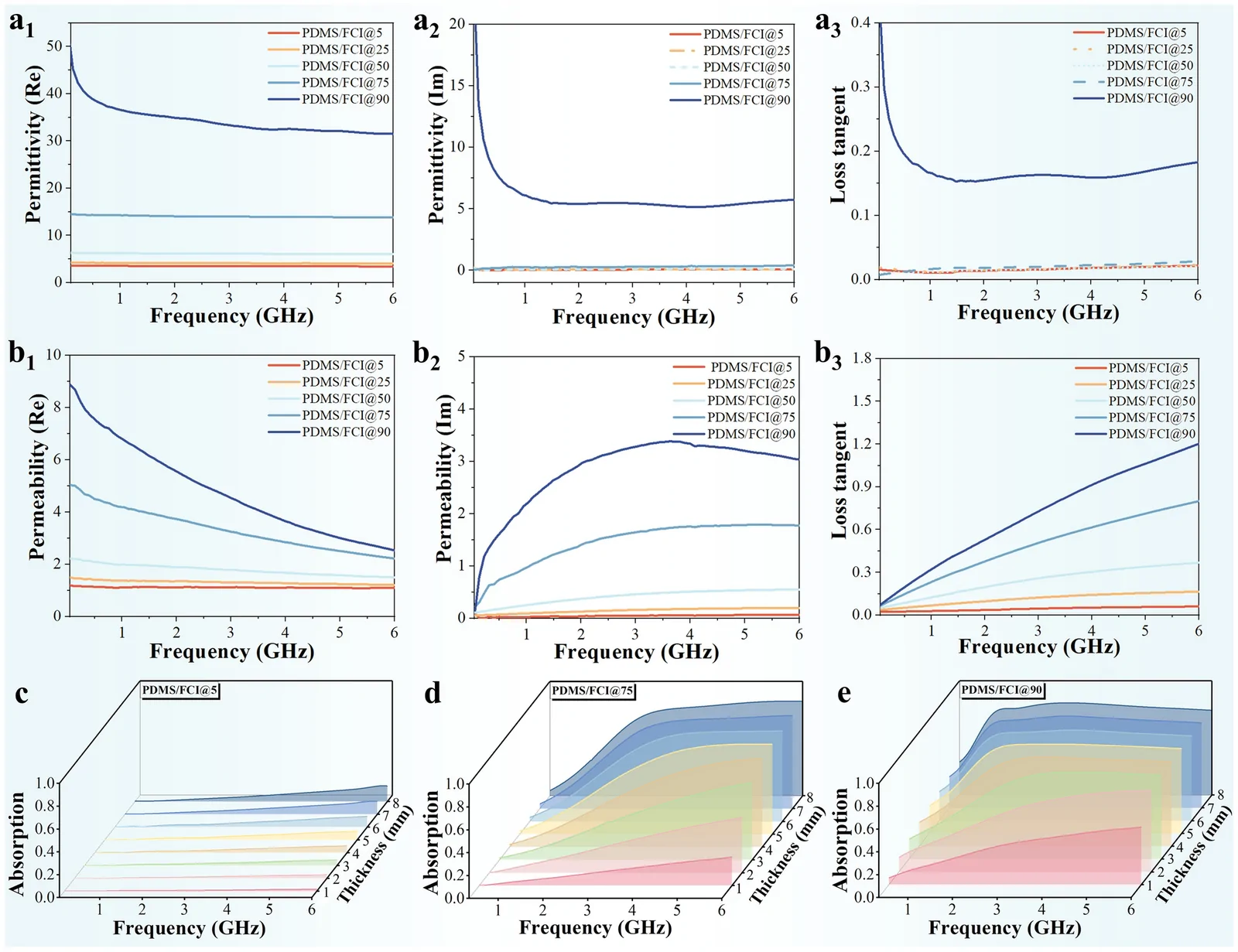
PDMS/FCI composites with different M-FCIs content: Dielectric properties:** a**_**1**_ Permittivity (Re), **a**_**2**_ Permittivity (Im), and **a**_**3**_ Loss tangent; Magnetic properties: **b**_**1**_ Permeability (Re), **b**_**2**_ Permeability (Im), and **b**_**3**_ Loss tangent; Absorption coefficient at thicknesses of 1–8 mm: **c** PDMS/FCI@5 composite, **d** PDMS/FCI@75 composite, and **e** PDMS/FCI@90 composite

The EM parameters of the composites were experimentally characterized using the coaxial-line method, and their absorption coefficient (*A*) over a thickness range of 1–8 mm was investigated via CST simulation (Fig. 2). The EM loss capacity of the PDMS/FCI composites is governed by the complex permittivity (*εᵣ* = *ε*′ − *jε*′′) and permeability (*μᵣ* = *μ*′ − *jμ*′′), whose frequency-dependent variations are presented in Fig. 2a, b. Both the real and imaginary parts of *εᵣ* and *μᵣ* exhibit a significant increase with higher M-FCIs content. The rise in complex permittivity is primarily attributed to enhanced dielectric polarization loss [27]. Higher particle concentrations create numerous micro‑capacitors at inter‑particle interfaces, leading to pronounced interfacial polarization relaxation (reflected in increased *ε*″). Furthermore, more extensive conductive networks above the percolation threshold contribute to greater conduction loss, resulting in notably elevated permittivity for the PDMS/FCI@90 composite. Simultaneously, the flaky shape of the carbonyl irons induces higher in-plane magnetic anisotropy, suppressing eddy‑current loss while promoting natural ferromagnetic resonance. The increased *μ*′ indicates enhanced magnetic energy storage, whereas increased *μ*′′ reflects more efficient magnetic energy dissipation from enhanced natural resonance and domain-wall motion in the densely packed magnetic composite [28, 29]. These results demonstrate that regulating the M‑FCIs content enables synchronous optimization of dielectric and magnetic losses across a broad frequency range, providing key material parameters for subsequent absorber design.

The S-parameters were calculated using CST Microwave Studio, and the corresponding absorption coefficient (*A* = 1 − *R* − *T*, where *R* is the reflection coefficient) over 1–8 mm thickness is displayed in Figs. 2c–e and S7. Although *A* increases with rising M‑FCIs content, the overall EM performance remains unsatisfactory for practical applications. Specifically, the *A* for all samples consistently does not exceed 0.9, and this limitation originates mainly from a significant impedance mismatch. That is, although a high M-FCIs content enhances EM loss, it also causes strong surface reflection due to excessively high complex permittivity and permeability, thereby hindering penetration and attenuation of incident waves inside the material.

### Design and Absorption Performance

**Fig. 3:**
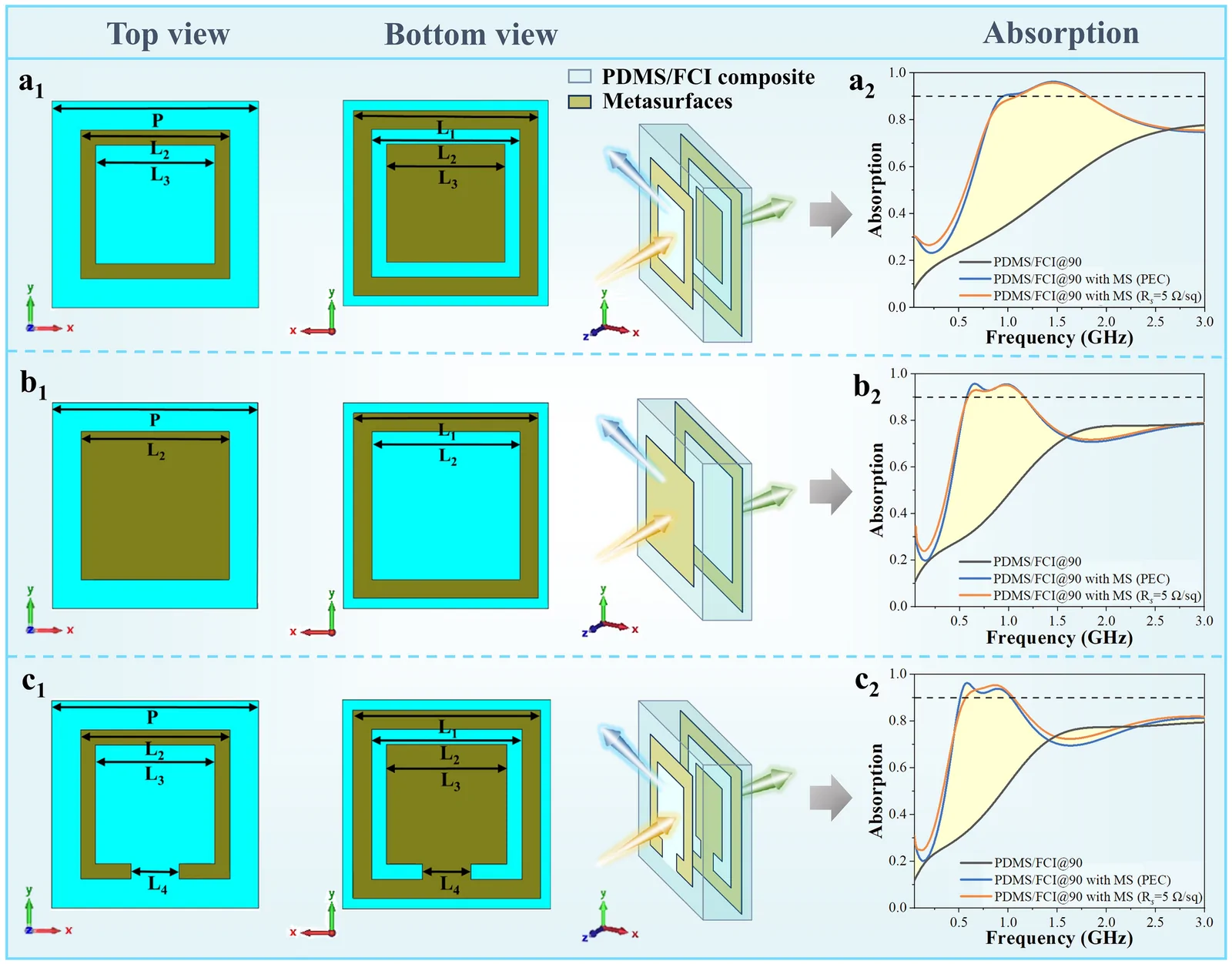
Schematic diagram of the CMFACs structure and comparison the absorption coefficient of CMFACs under different parameter designs: **a** Square-ring MS, **b** Square MS, and **c** Square open-ring MS

As shown in Fig. 2c, the LF EM absorption performance is generally poor at small material thicknesses. To overcome this limitation, an MS structure was integrated onto the M‑FCIs absorbing medium to enhance LF absorption. MS are artificially engineered arrays of subwavelength periodic units whose EM properties are primarily determined by their geometry rather than composition [30, 31]. These structures can resonate at specific frequencies, effectively coupling and localizing EM field energy to generate a highly enhanced localized field, with amplitudes potentially reaching tens of times that of the incident field. This strong localized field interacts with the adjacent absorbing medium, markedly improving its capacity to capture and dissipate EM energy. Accordingly, a double‑layer MS configuration composed of complementary unit arrays was designed (Detailed geometries in Fig. 3a_1_, b_1_, and c_1_). The interlayer spacing is much smaller than the operating wavelength, enabling strong near‑field coupling both within and between the two MS layers. Tuning structural parameters (relative positioning, spacing, dimensions) optimizes the coupling strength and resonant characteristics are optimized, improving the overall EM response and its synergy with the magnetic substrate for enhanced LF absorption.

Taken together, with the EM parameters of the PDMS/FCI@90 composite used as the lossy substrate for CMFAC, based on A within the 0.3–3.0 GHz as the evaluation criterion, the periodic structure parameters, the material properties of the MS layer, and the substrate thickness were systematically optimized via the Genetic Algorithm built into CST Microwave Studio. This algorithm generates points within the parameter space and progressively converges toward the global optimum through multi‑generational random mutation and selection of the “fittest” parameter sets, making it well-suited for complex problem domains and models with numerous parameters. The detailed settings of the optimizer are shown in Tables S2 and S3, and the corresponding optimization results are presented in Table S5. From Fig. 3a_2_, b_2_, and c_2_, the incorporation of the double-layer coupled MS unit arrays led to a significant enhancement in the *A* value of the CMFAC without an obvious decline as frequency increased. At a total thickness of 3.78 mm, the CMFAC achieved effective absorption within the 0.95–1.81 GHz band. For thicknesses of 5.59 and 6.16 mm, the effective absorption bands were 0.57–1.16 and 0.51–1.03 GHz, respectively. Furthermore, the absorption performance was compared for MS layers modeled as a PEC versus a resistive film with a *Rₛ* of 5 Ω sq^−1^. Simulations indicated that the *A* value of the designed CMFAC using the resistive film (*Rₛ* = 5 Ω sq^−1^) was comparable to that using the PEC. Notably, when the mass fraction of M-FCIs was relatively low (below 75%), the performance of the resistive film even surpassed that of the PEC, as shown in Fig. S8. In addition, the absorption performance under TE and TM polarizations across incident angles from 0° to 80° within the 0.3–3.0 GHz confirms that the designed CMFAC exhibits outstanding angular stability. The difference in absorption performance between TE and TM polarizations under oblique incidence originates from the asymmetric EM response of the MS to electric and magnetic fields. For TE polarization, the electric field is parallel to the plane of MS plane. Under oblique incidence, the magnetic field possesses a component perpendicular to the MS plane, which effectively excites the magnetic resonance of the square-ring structure. In this case, both electric resonance and magnetic resonance coexist, and their mutual coupling influences the overall absorption performance. In contrast, for TM polarization, the magnetic field is parallel to the plane of MS plane. Under oblique incidence, the magnetic field has no perpendicular component to the MS plane, thus primarily exciting electric resonance alone. The structure maintains excellent absorption efficiency within 50° for TE polarization and within 70° for TM polarization (Fig. S9). This robust angular stability can be attributed to the effective excitation of local field enhancement by the CMFAC for both polarization states.

### Field Distribution and Loss Mechanism Analysis

**Fig. 4:**
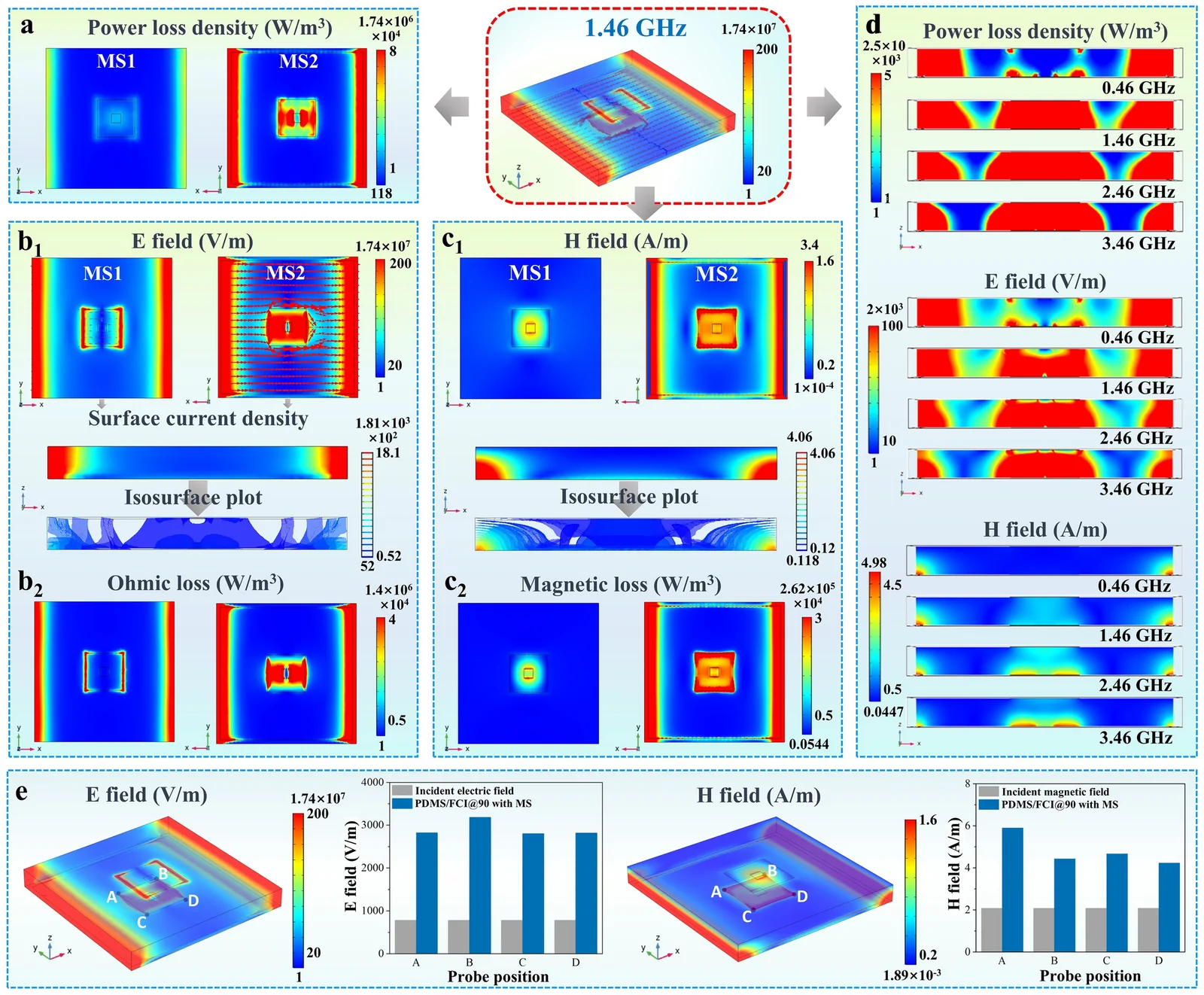
Field distribution of the CMFAC based on square-ring MS under TE polarization: **a-c** Surface view at 1.46 GHz; **d** Section views at 0.46 GHz, 1.46 GHz, 2.46 GHz, and 3.46 GHz; **e** Comparison of the electric field and magnetic field intensities with the corresponding initial incident intensities at 1.46 GHz

It should be noted that while the three structures studied herein display distinct resonant frequencies and absorption bandwidths, they all operate based on the same underlying physical mechanism—namely local field enhancement and multimode coupling enabled by the interaction between double-layer coupled MS and the magnetic substrate. Accordingly, the CMFAC employing the square-ring MS is chosen as a representative configuration for a detailed mechanistic analysis in the main text. The coupling enhancement between the localized fields of double-layer MSs at subwavelength spacings and EM dissipation in the designed CMFACs was further investigated via COMSOL Multiphysics simulations, and the corresponding spatial distributions of the electric field, magnetic field, magnetic loss, surface current, and power loss density are presented in Figs. 4 and S10. The isosurface plots in Fig. 4b_1_ and c_1_ clearly illustrate the 3D distributions of the electric and magnetic fields. The isosurfaces between the double-layer MS are interconnected, indicating that due to EM induction, a magnetic field penetrating both MS layers is induced. This field couples with the magnetic substrate, significantly enhancing its absorption. This interconnection confirms that both MS layers act as coupled resonators, and their strong near‑field interaction at deep‑subwavelength spacing leads to significant field localization and amplification, enhancing energy dissipation. Specifically, the upper layer couples incident energy, exciting a circulating current and generating a magnetic dipole moment. This moment transfers to the lower layer via near-field induction, creating a strong magnetic field in the gap. The resulting surface current concentrates at the inner corners and edges of the lower MS, converting energy into heat via eddy-current loss. The electric and magnetic fields in these corners have been drastically enhanced, exceeding the incident strength. As shown in Fig. 4e, the electric field intensity increases nearly threefold, and the magnetic field intensity nearly fourfold, confirming the key role of magnetic near‑field enhancement in activating substrate loss. Notably, the oppositely directed currents in the two layers produce counter-rotating magnetic dipoles (Fig. 4b_1_) that cancel in the far field [32, 33], suppressing far-field reflection and scattering, and localizing energy within the structure [34]. This field-loss co-design enables efficient energy coupling, transfer, and conversion, significantly boosting the overall absorption efficiency of the CMFAC.

Although the three MS structures are based on the same physical mechanism, they exhibit significant differences in resonant frequency and absorption bandwidth, which stem from the distinct modulation of EM field distributions and surface current states by their respective geometries. For the CMFAC based on the square-ring MS (Fig. 4b, c), the electric field is distributed along both the inner and outer edges of the rings, while the magnetic field forms circulating loops within the ring apertures, establishing a distributed LC resonance. In this configuration, the inductance primarily originates from the ring-shaped conductive paths, and the capacitance arises from the coupling between the upper and lower MS layers. The relatively balanced contributions of inductance and capacitance result in a resonant frequency of 1.46 GHz. In terms of the surface current distribution, the currents at both ends of the square-ring flow in the same direction. There are two dipoles present, and the interaction between these two dipoles causes a broadening of the resonance peak. For the CMFAC based on the square MS (Fig. S10a_2_ and a_3_), the electric field is strongly concentrated at the edges of the square patterns. Compared to the square-ring MS, this edge-concentrated field distribution gives rise to a significantly increased equivalent capacitance. The larger capacitance dominates the LC product, thereby shifting the resonant frequency down to 0.65 GHz. In terms of the surface current distribution, only a single dipole exists within the square structure, forming a single resonant current path, which results in a narrower resonance peak. For the CMFAC based on the square open-ring MS (Fig. S10b_2_ and b_3_), the lowest resonant frequency (0.58 GHz) is achieved. This can be attributed to the following factors. First, the gap in each open ring forms a localized capacitive hotspot, where the electric field is intensely concentrated, substantially increasing the equivalent capacitance. Second, the symmetry breaking of the open ring introduces an additional gap capacitance into the resonant circuit, further enhancing the total capacitance. These combined capacitive effects result in the largest equivalent capacitance among the three structures, thereby yielding the lowest resonant frequency. In terms of the surface current distribution, a large dipole exists within the square open ring, with a current path length approximately three times that of the other two structures. This extended current path contributes to the reduced resonant frequency while also narrowing the resonance peak.

### Experimental Characterization

**Fig. 5:**
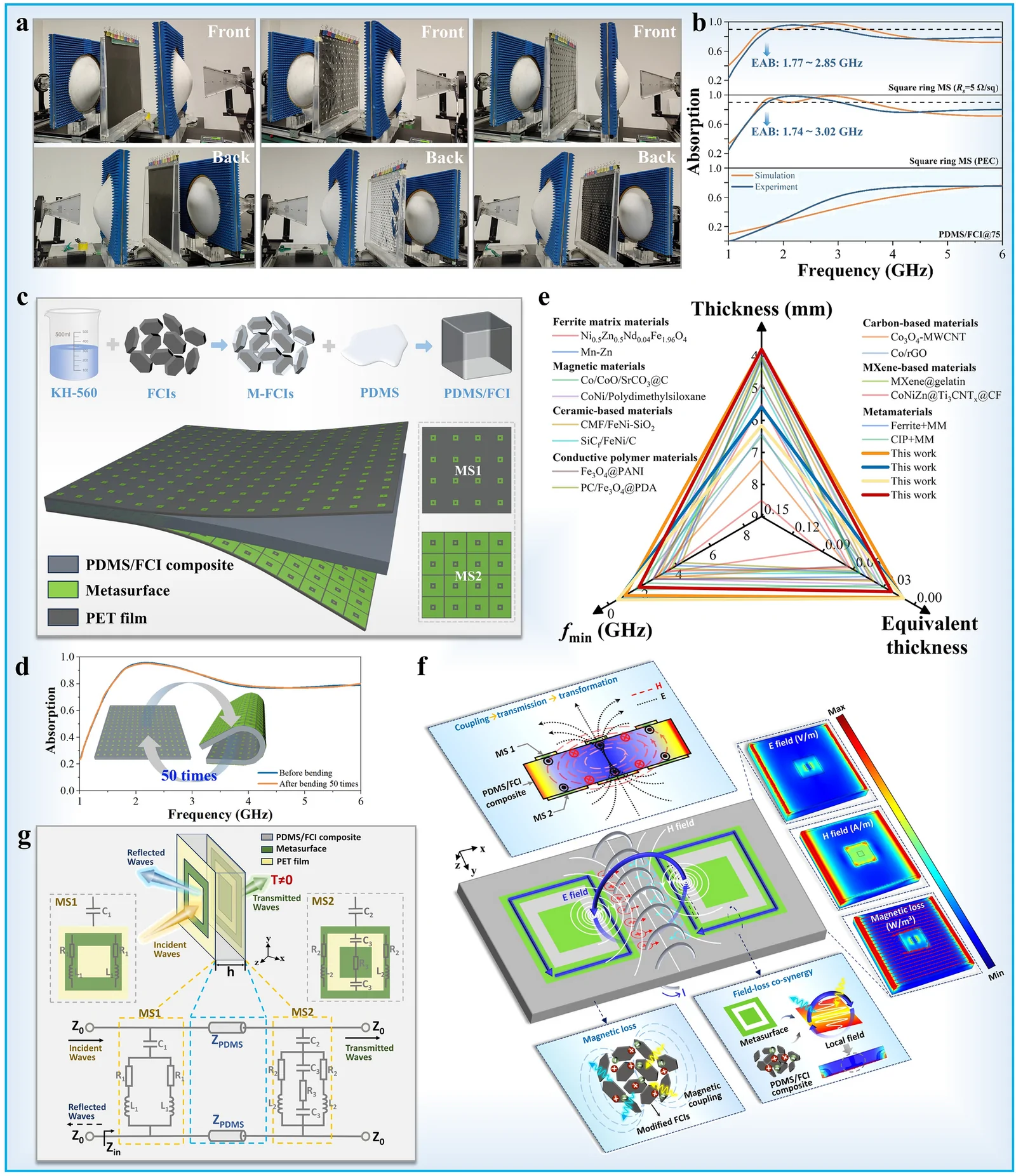
Experimental validation and mechanism analysis:** a** Photos of the free-space measurement site (from left to right): PDMS/FCI@75 composite, PDMS/FCI@75 composite with square-ring MS (Ni film), and with square-ring MS (PEDOT:PSS film, *Rₛ* = 5.84 Ω sq^−1^); **b** Comparison of simulated and experimental absorption coefficients; **c** Flowchart of the preparation of PDMS/FCI composite and structural schematic of the CMFAC; **d** Absorption coefficient of the CMFAC before and after cyclic bending; **e** Performance comparison with 14 representative LF absorbers; **f** Schematic of the EM absorption mechanism; **g** Equivalent circuit diagram

To verify the simulation structure and investigate the synergistic enhancement between the MS localized field and EM loss, a PDMS/FCI@75-based MS composite (500 mm × 500 mm × 3.78 mm) was fabricated according to the dimensions shown in Fig. 4a. The measured A for the bare substrate, as well as for the Ni‑based MS and PEDOT:PSS‑based MS structures, incorporated into the CMFAC, is shown in Fig. 5a. The experimental results agree well with simulations (Fig. 5b). After MS integration, A exceeds 0.9 in the 1.77–2.85 GHz, representing up to a 165% improvement, confirming the effectiveness of the MS-enhanced LF absorption via localized field enhancement. The nearly identical performance of PEDOT:PSS and Ni‑based MS demonstrates design compatibility, and the minor deviations between the experimental and simulation results stem from fabrication tolerances, such as dispersion uniformity, thickness variation, machining accuracy, and so on. In response to the typical application requirements of CMFAC on flexible skins, we tested the wave absorption performance after 50 bending cycles. Owing to the inherent flexibility and durability of the PDMS/FCIs@75 composite and the PEDOT:PSS film, the CMFAC maintains stable EM wave absorption performance after 50 bending cycles (Fig. 5c, d), exhibiting integrated flexibility and excellent structural stability. Figure 5e compares the actual thickness, equivalent thickness, and the lowest effective absorption frequency (*f*_min_) of the CMFAC with representative LF absorbers [35–48]. More detailed quantitative comparisons are provided in Table S6, indicating that the proposed design achieves superior absorption performance at a reduced overall thickness.

Figure 5f elucidates the physical absorption mechanism of the CMFAC structure, arising from synergistic localized‑field enhancement and multimode loss, including the magnetic dipole resonance between MS layers, hysteresis loss in the substrate, and field‑loss interaction. Incident energy is first coupled by the top MS, partially dissipated via ohmic and eddy‑current losses. The remainder enters the magnetic substrate, where enhanced magnetic coupling promotes natural resonance, efficiently attenuating the magnetic component [49, 50]. Strong interlayer EM coupling induces high‑density circulating currents, forming “magnetic hot spots” with the substrate [51, 52]. This localized field significantly increases the polarization relaxation rate and hysteresis loop area of magnetic particles, synergistically enhancing both dielectric and magnetic losses. Therefore, the integrated CMFAC can achieve a 165% increase in *A*. To further quantitatively analyze this cooperative loss mechanism and establish its generalized relationship with structural parameters, an equivalent circuit model for the CMFAC is presented in Fig. 5g. The MS layer is represented by equivalent resistance (*R*), inductance (*L*), and capacitance (*C*), while the PDMS/FCI@75 substrate is modeled as a transmission line (impedance *Z*_PDMS_) providing phase delay and impedance transformation. Overlapping absorption peaks are achieved by cascading two RLC branches with distinct resonant frequencies [53, 54]. Tuning the MS parameters aligns these resonances with the substrate’s natural frequency, merging the peaks into a continuous broadband. The input impedance and admittance are given by:

1 $$Z_{{{\mathrm{FSS}}1}} = \frac{1}{{j\omega C_{1} }} + \frac{1}{{\frac{2}{{R + j\omega L_{1} }}}}$$

2 $$Y_{{{\mathrm{FSS}}1}} = \frac{{2R - j\left( {\omega L_{1} C_{1} - \frac{4}{{\omega C_{1} }}} \right)}}{{R^{2} + \left( {\omega L_{1} - \frac{2}{{\omega C_{1} }}} \right)^{2} }}$$

3 $$Z_{{{\mathrm{FSS}}2}} = \frac{1}{{j\omega C_{2} }} + \frac{1}{{\frac{2}{{R + j\omega L_{2} }} + \frac{1}{{\frac{2}{{j\omega C_{3} + R}}}}}}$$

4 $$Y_{{{\mathrm{FSS}}2}} = \frac{{\omega C_{2} \left( {5R + j\omega L_{2} + 4j\omega C_{3} } \right)(\omega \left( {4C_{3} + L_{2} } \right) + 2\omega C_{2} \left( {R - \omega^{2} C_{3} L_{2} } \right) - jR\left( {2C_{2} \omega^{2} \left( {C_{3} + L_{2} } \right) - 5} \right)}}{{\left( {\omega \left( {4C_{3} + L_{2} } \right) + 2\omega C_{2} \left( {R^{2} - \omega^{2} C_{3} L_{2} } \right)} \right)^{2} + \left( {R\left( {2C_{2} \omega^{2} \left( {C_{3} + L_{2} } \right) - 5} \right)} \right)^{2} }}$$

where Z_FSS1_ and Z_FSS2_ present the equivalent impedance of the MS layers, Y_FSS1_ and Y_FSS2_ present the equivalent admittance, *ω* is the angular frequency, *R* is the equivalent resistance, *L* is the equivalent inductance, and *C* is the equivalent capacitance.

### Radar Cross Section Simulation Analysis

**Fig. 6:**
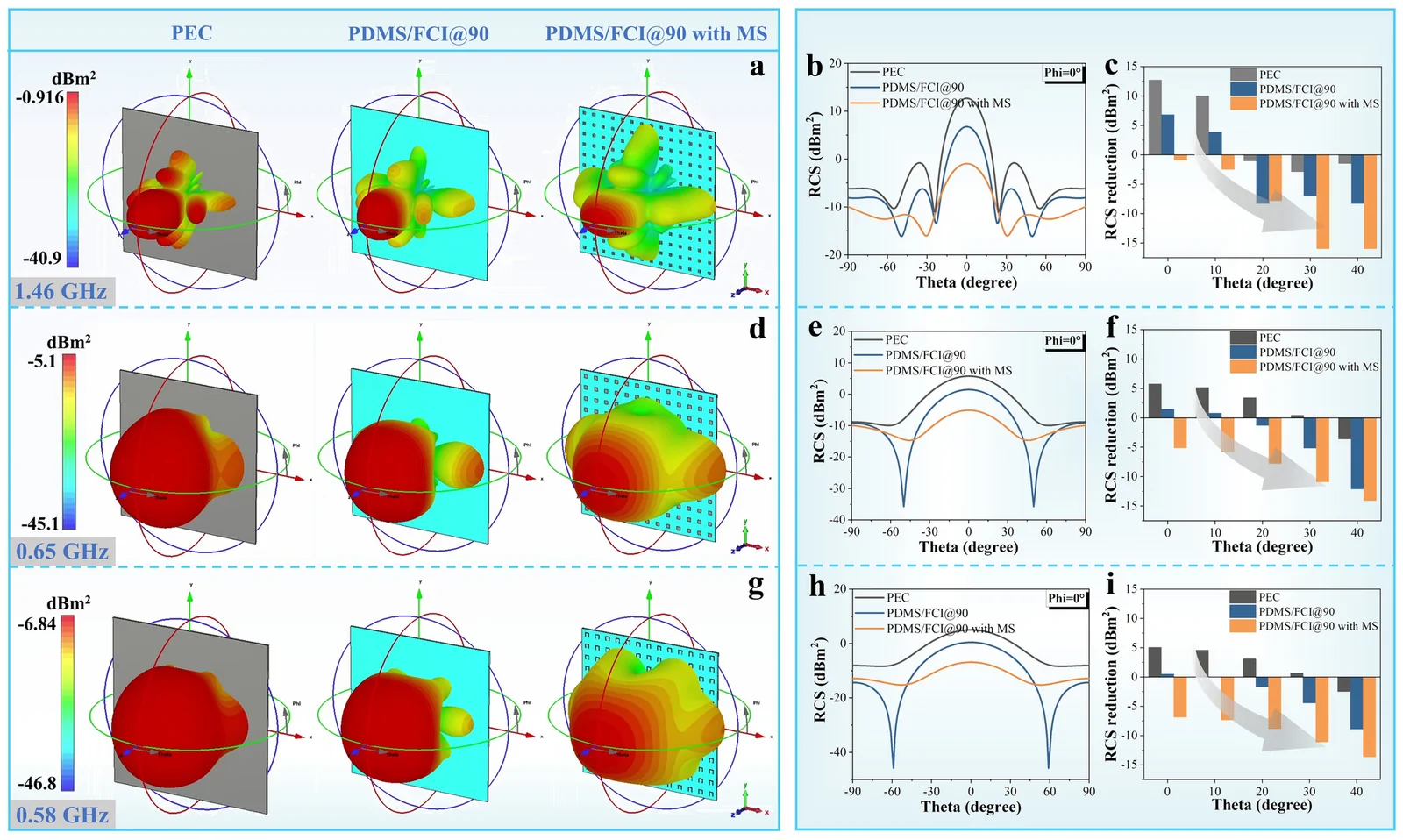
Simulated RCS performance of CMFACs with different MS unit cells: **a**–**c** square-ring, **d**–**f** square, and **g**–**i** square open ring. For each configuration, the panels present **a, d, g** 3D far-field patterns, **b, e, h** RCS plots in Cartesian coordinates at specific detection angles, and **c, f, i** corresponding RCS reduction values

To further evaluate the engineering potential of the CMFAC, the radar cross section (RCS) of a standard 500 mm × 500 mm plate was simulated over an incidence angle range of − 90° to 90° (Figs. 6 and S11). The 3D RCS plots (Fig. 6a, d, and g) demonstrate that all MS structures achieve significant RCS reduction, and the RCS of square-ring based CMFAC at 0° is reduced by > 10 dB relative to the PEC and by > 7 dB relative to the PDMS/FCI@90 composite without an MS structure at 1.46 GHz, confirming the substantial contribution of MS structures to LF absorption. Furthermore, the design demonstrates excellent capability in sidelobe suppression, and the sidelobe energy within ± 40° is also decreased by about 10 dB (Fig. 6b, c), demonstrating omnidirectional RCS reduction capability.

### Application Simulation and Analysis

**Fig. 7:**
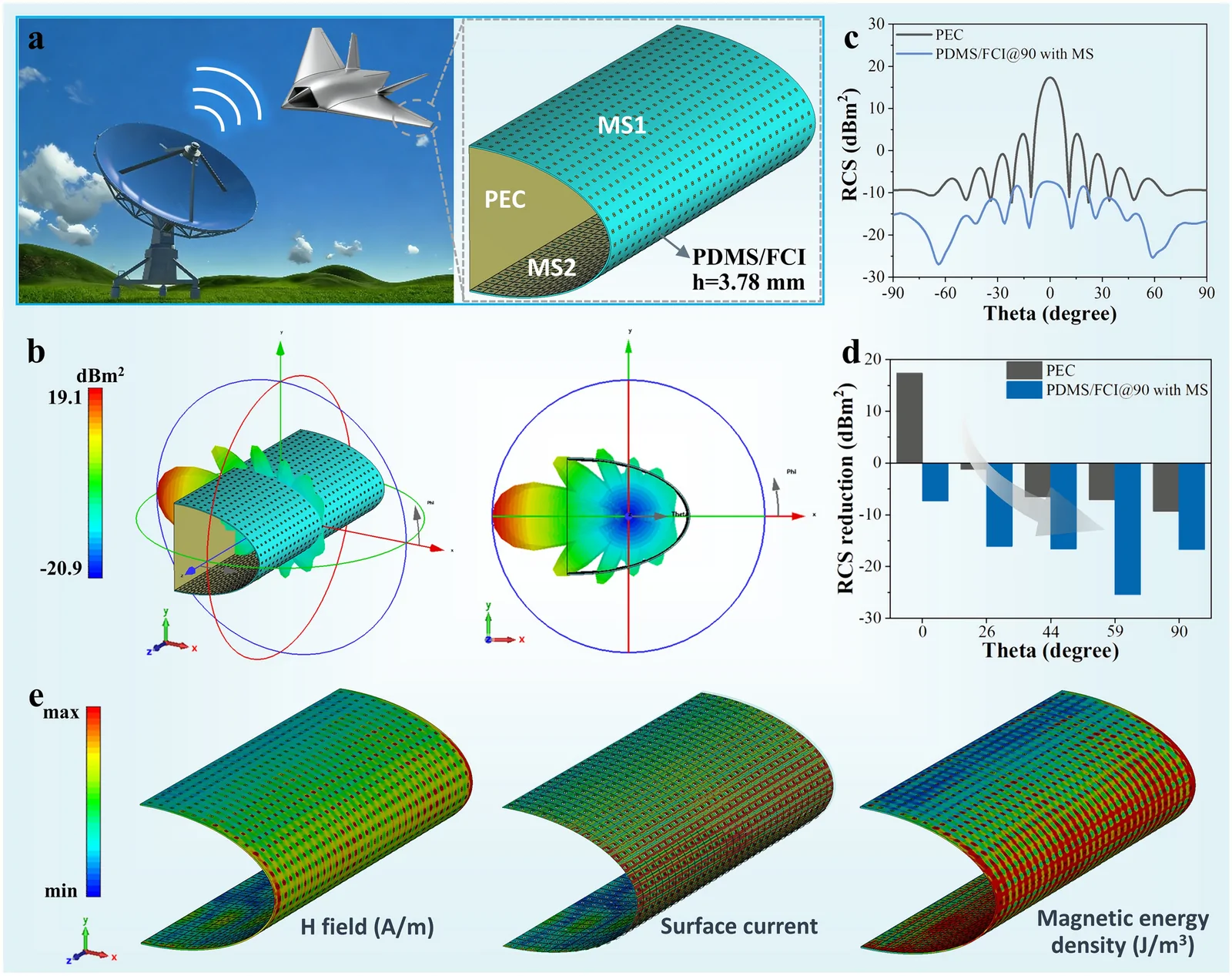
CMFAC with square-ring MS: **a** Application scenarios; **b** 3D far-field diagram; **c** RCS plot in the Cartesian coordinate system under certain detecting angles; **d** RCS reduction values; **e** Field distribution

To verify feasibility in practical engineering, an aircraft leading-edge structure was extracted, and its surface was covered with CMFAC (Fig. 7a). The leading edge (length 2 m, bottom width 600 mm, and height 400 mm) was conformally coated with a 3.78-mm‑thick PDMS/FCI substrate, with MS on the upper and lower surfaces and a PEC sheet at the bottom. Monostatic RCS in the ± 90° azimuth range under VV polarization was simulated, using a metal plate of the same bottom size as a reference. The simulation results indicate that the monostatic RCS of the leading-edge structure covered with conformal CMFAC is significantly lower than that of the metal plate within the ± 90° azimuth angle range, especially at the 0° incidence direction (normal incidence), where the RCS reduction reaches up to 24.75 dB (Fig. 7c, d). The far‑field scattered energy distribution at 0° incidence, as shown in Fig. 7b, indicates strong attenuation along the incident direction (-x axis). As shown in Fig. 7e, the magnetic field, surface current, and magnetic field intensity on the conformal CMFAC surface at 0° incidence reveal obvious local‑field characteristics, including the MS units strongly coupling the magnetic field, concentrating magnetic energy near the structure, and significantly enhancing field intensity and energy density. The surface current excited by the incident wave decays gradually during transfer from top to bottom, greatly reducing RCS intensity. These results demonstrate that the designed CMFAC maintains excellent wide‑angle absorption even on high‑curvature surfaces, solving the performance degradation problem of traditional absorbers on complex curved surfaces and showing great potential for next‑generation conformal stealth technology.

## Conclusions

This study addresses the fundamental trade-off between ultra-thin thickness and broadband absorption in LF absorbers by designing, fabricating, and experimentally validating an ultra-thin, flexible, and cascaded P-band MS absorber. A novel “localized EM field enhancement” strategy, which utilizes a flexible PDMS/FCI composite as the magnetic substrate with coupled MS layers integrated on its upper and lower surfaces is proposed. Further, the near‑field coupling between the MS layers enables the generation of significantly enhanced, highly localized EM fields within the magnetic substrate for efficient conversion and dissipation of incident energy. By replacing the conventional metal backplane with a functional, transmissive MS, we overcome the thickness-to-wavelength constraint, permit non-zero transmission, and establish a foundation for vertical cascading. At a total thickness of 3.78 mm, CMFAC achieves an absorption rate exceeding 90% across 1.77–2.85 GHz, demonstrating outstanding LF absorption performance with ultra‑thin thickness. The use of a PEDOT:PSS-based MS provides performance comparable to metal-based structures while enabling a pathway toward lightweight, conformal applications. Excellent mechanical resilience under cyclic stress ensures reliable integration on complex curved surfaces. Therefore, this study introduces a novel “field-loss co-synergy” paradigm, offering a feasible and scalable route to advanced absorptive materials that combine LF stealth, flexibility, and system integration.

## Supplementary Information

Below is the link to the electronic supplementary material.

Supplementary file1 (DOCX 42018 KB)
