# Supplementary material for: Cascaded Broadband Low-Frequency Microwave Absorption Covering P- to C-Band in Ultra-Thin Metamaterials via Synergistic Local‑Field and Loss‑Field Enhancement
Source: Nanomicro Lett. 2026 Jun 22;18:407. doi: 10.1007/s40820-026-02245-1 (PMC13287531; doi:10.1007/s40820-026-02245-1)
Supplement: Supplementary file 1 — Supplementary file1 (DOCX 42018 KB) [file 40820_2026_2245_MOESM1_ESM.docx]

Supporting Information for

**Cascaded Broadband Low-Frequency Microwave Absorption covering P- to C-Band in Ultra-Thin Metamaterials via Synergistic Local‑Field and Loss‑Field Enhancement**

Qian Yang^1^, Hongbo Hou^1^, Yongxi Lu^1^, Zhongqiu Guo^1^, Jiaxu Sun^1^, Peng Zhang^1^, Tian Yang^1^*, and Fanbin Meng^1^*

^1^ Key Laboratory of Advanced Technologies of Materials (Ministry of Education), School of Materials Science and Engineering, Southwest Jiaotong University, Chengdu 610031, P. R. China

*Corresponding authors. E-mail: [yangtian509@163.com](mailto:yangtian509@163.com) (Tian Yang); [mengfanbin_wing@126.com](mailto:mengfanbin_wing@126.com) (Fanbin Meng)

**S1 Details of SEM image of FCIs and PDMS/FCI composites**

**
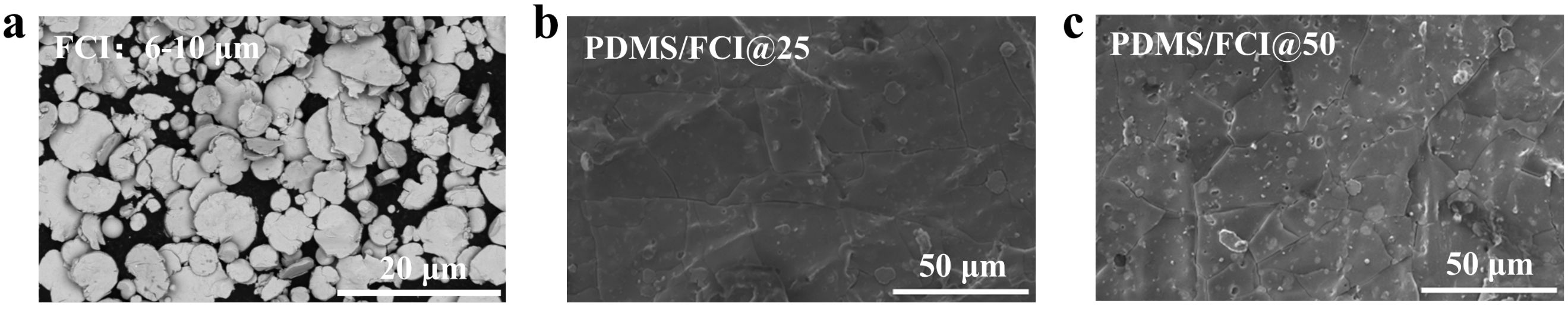
**

**Fig. S1** SEM image: **a** FCIs; **b** PDMS/FCI@25 composite; **c** PDMS/FCI@50 composite.


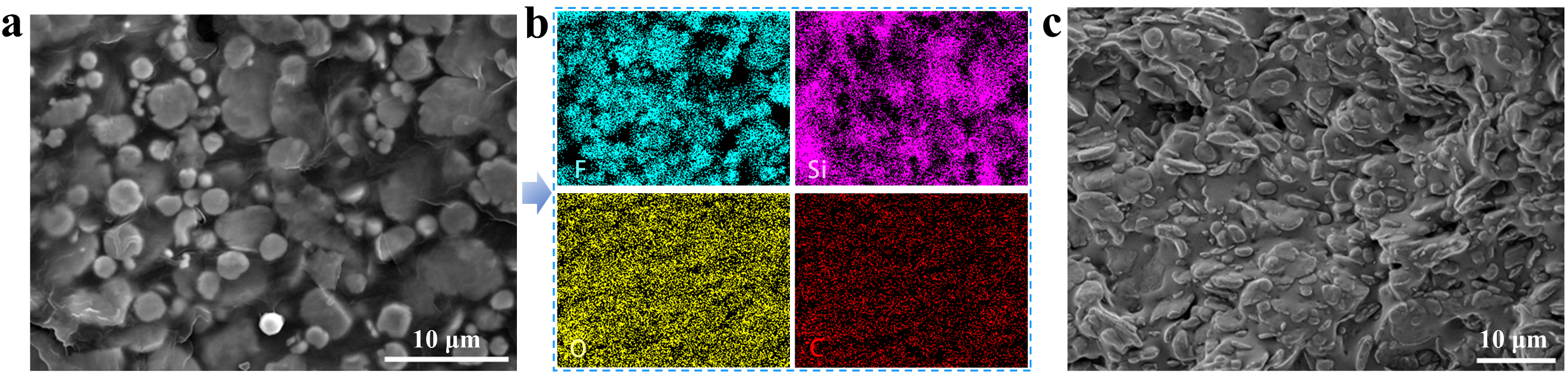


**Fig. S2.** PDMS/FCI@75 composite: **a** Surface SEM image; **b** EDS mapping distribution; **c** SEM image of the tensile fracture surface.

**S2 Details of the mechanical properties of PDMS/FCI composites**

**
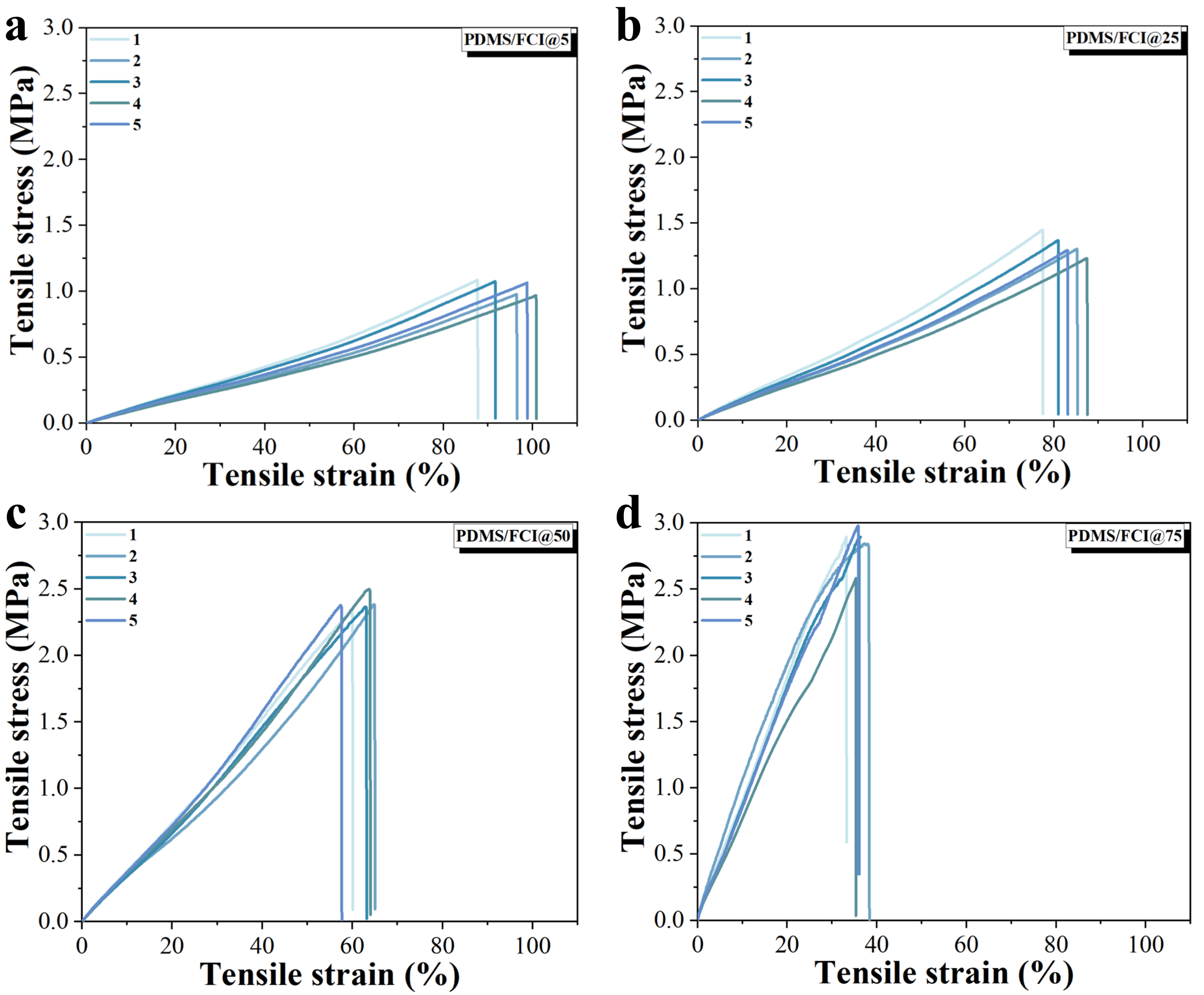
**

**Fig. S3** Tensile stress-strain curve: **a** PDMS/FCI@5 composite; **b** PDMS/FCI@25 composite; **c** PDMS/FCI@50 composite; **d** PDMS/FCI@75 composite.

**
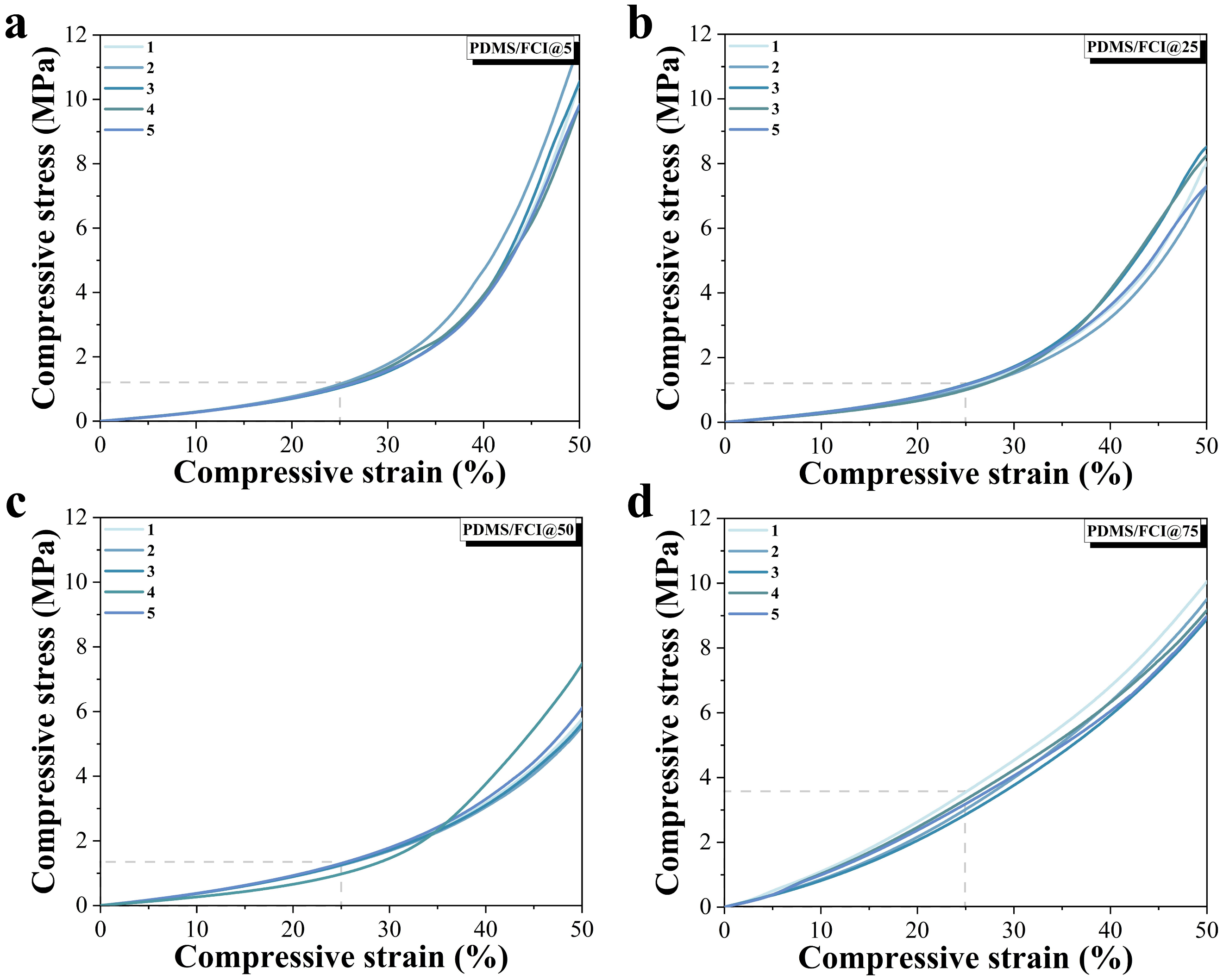
**

**Fig. S4** Compression stress-strain curve: **a** PDMS/FCI@5 composite; **b** PDMS/FCI@25 composite; **c** PDMS/FCI@50 composite; **d** PDMS/FCI@75 composite.

**
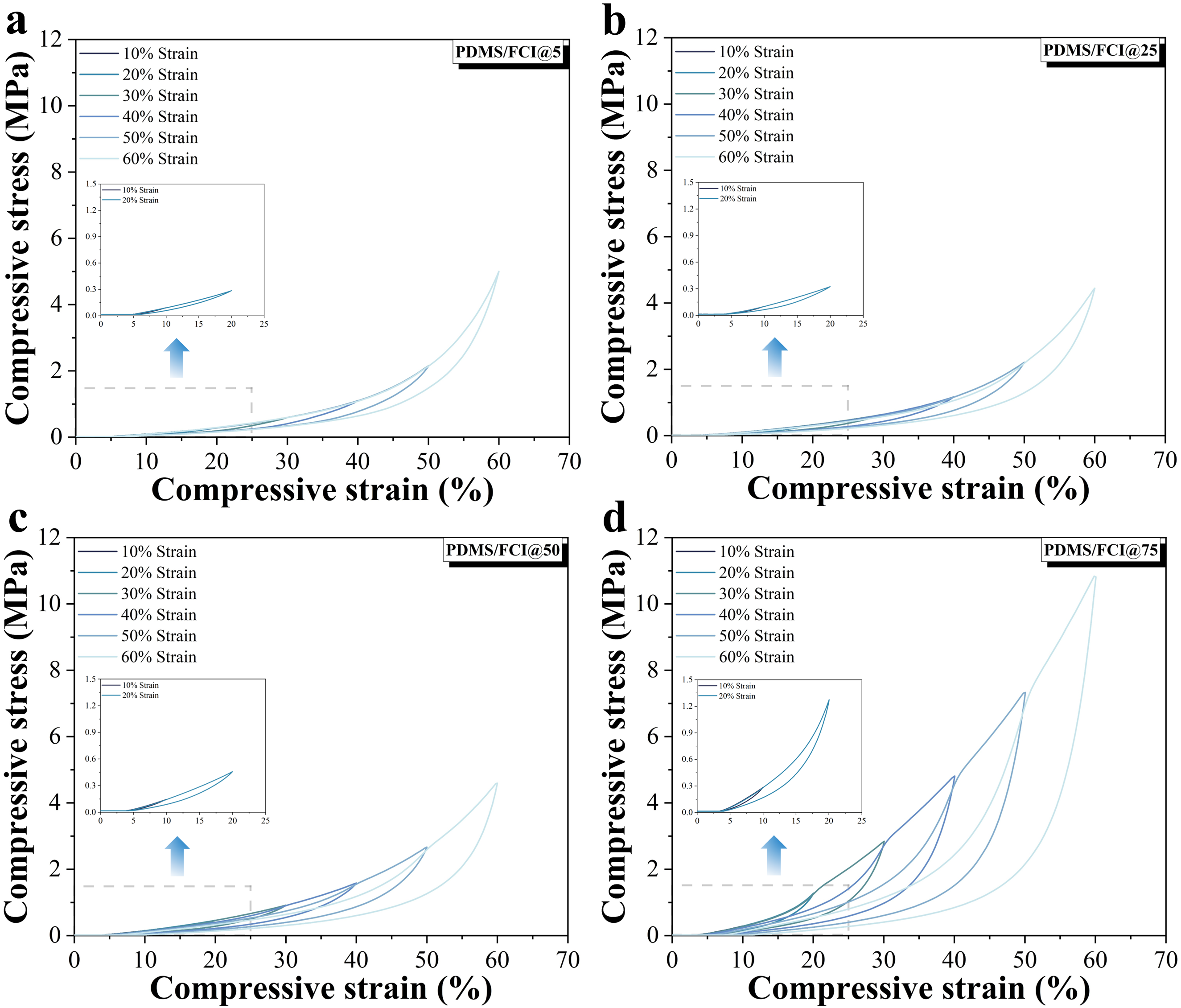
**

**Fig. S5** Compression stress-strain curve at 10-60% strain: **a** PDMS/FCI@5 composite; **b** PDMS/FCI@25 composite; **c** PDMS/FCI@50 composite; **d** PDMS/FCI@75 composite.

**S3 Details of the KH-560 modified FCIs**

In this study, a pristine FCIs were modified with the KH-560, aiming to enhance interfacial compatibility and dispersion uniformity within the PDMS matrix. The success of surface functionalization was further confirmed by Fourier transform infrared (FTIR) spectroscopy. As shown in Fig S6a, M-FCIs exhibit new characteristic absorption peaks compared to unmodified FCIs. Specifically, the peak at 822 cm^-1^ corresponds to the Si-O-CH_3_ vibrational absorption. Notably, the broad peak around 1088 cm⁻¹ is assigned to the asymmetric stretching vibration of the cross-linked Si-O-Si network, overlapping with Si-O-C from KH-560. Furthermore, the peak at 1270 cm^-1^ indicates the ring-symmetric stretching vibration of the epoxy group in the organic moiety, confirming that the organic moiety from KH-560 remain intact on the FCIs surface to form an active coating layer. This enhanced interfacial compatibility is crucial for mitigating particle agglomeration, ensuring uniform filler distribution, and ultimately optimizing the EM and mechanical properties of the final composite.

To macroscopically evaluate the overall surface elemental composition, XPS survey scanning was first conducted (Fig S6b). As illustrated, the survey spectrum of M-FCIs exhibits the characteristic base peaks of Fe 2p (~711 eV), O 1s (~531 eV), and C 1s (~285 eV). More importantly, a distinct new peak emerges at approximately 102 eV, which is assigned to Si 2p. Considering the absence of silicon in the pristine FCIs, the emergence of these Si signals provides direct and intuitive macroscopic evidence for the successful attachment of the KH-560 onto the surface of the FCIs, laying a preliminary foundation for the subsequent high-resolution mechanistic analysis.

As illustrated in Fig. S6c, the oxidation kinetics and thermal stability were evaluated via TG and DTG curves. Due to the high reactivity of iron, both FCIs and M-FCIs exhibited a general weight gain at elevated temperatures under N₂ atmosphere (trace oxygen effect). Notably, in the range of 350-530℃, M-FCIs showed a suppressed initial weight gain compared to FCIs, which is attributed to the physical barrier effect provided by the dense polysiloxane network that inhibits oxygen diffusion. Subsequently, a more pronounced DTG downward peak for M-FCIs were observed between 535-580℃, assigned to the thermal cleavage of the grafted silane chains. These findings are in good agreement with the XPS results, collectively confirming the successful chemical grafting of KH-560 and its protective role for the iron core.


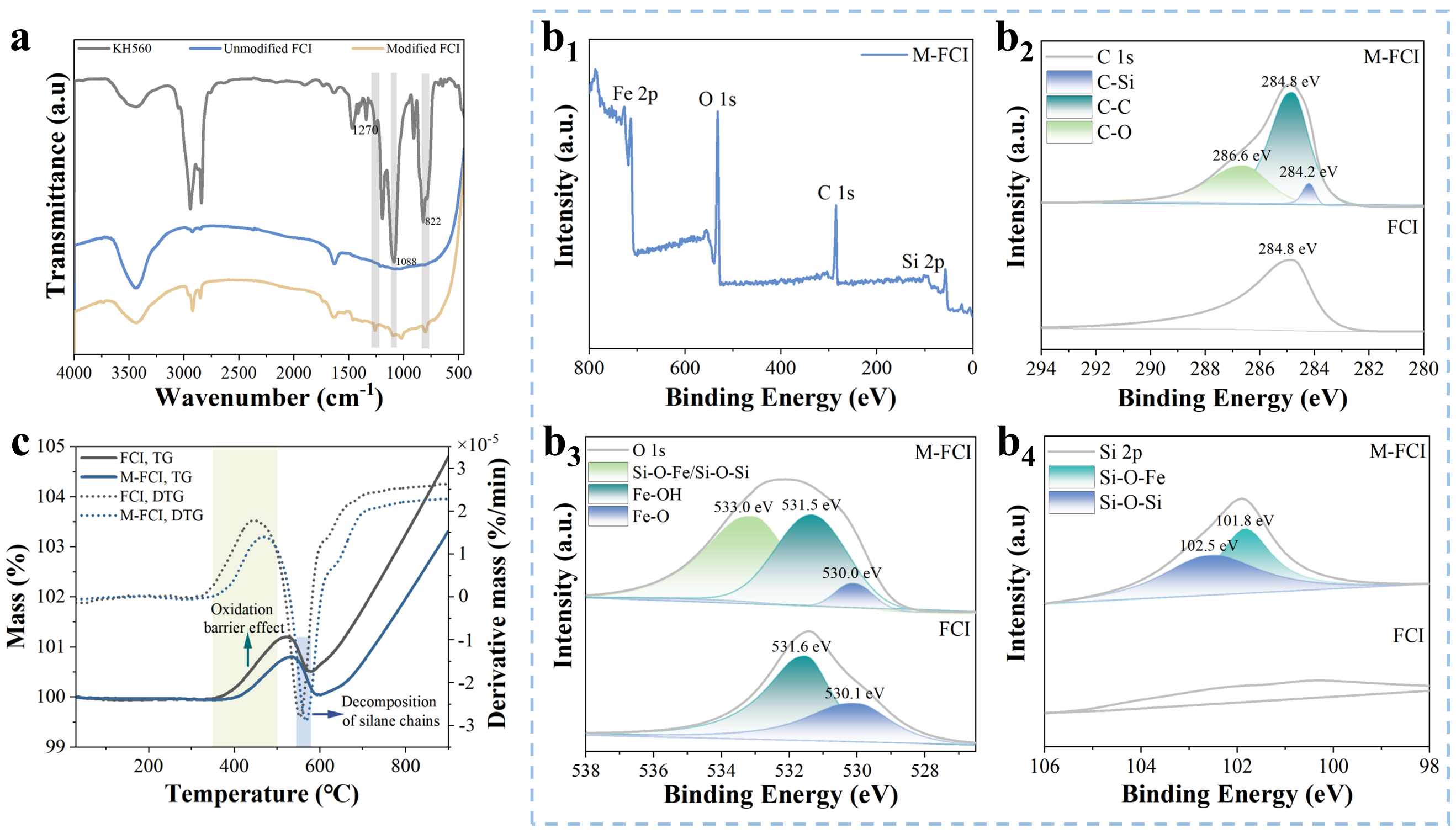


**Fig. S6** FCIs before and after modification with KH-560: **a** FTIR spectra; **b** XPS spectra; **c** TG and DTG curves.

**S4 Details of the absorption coefficient of PDMS/FCI composites**

**
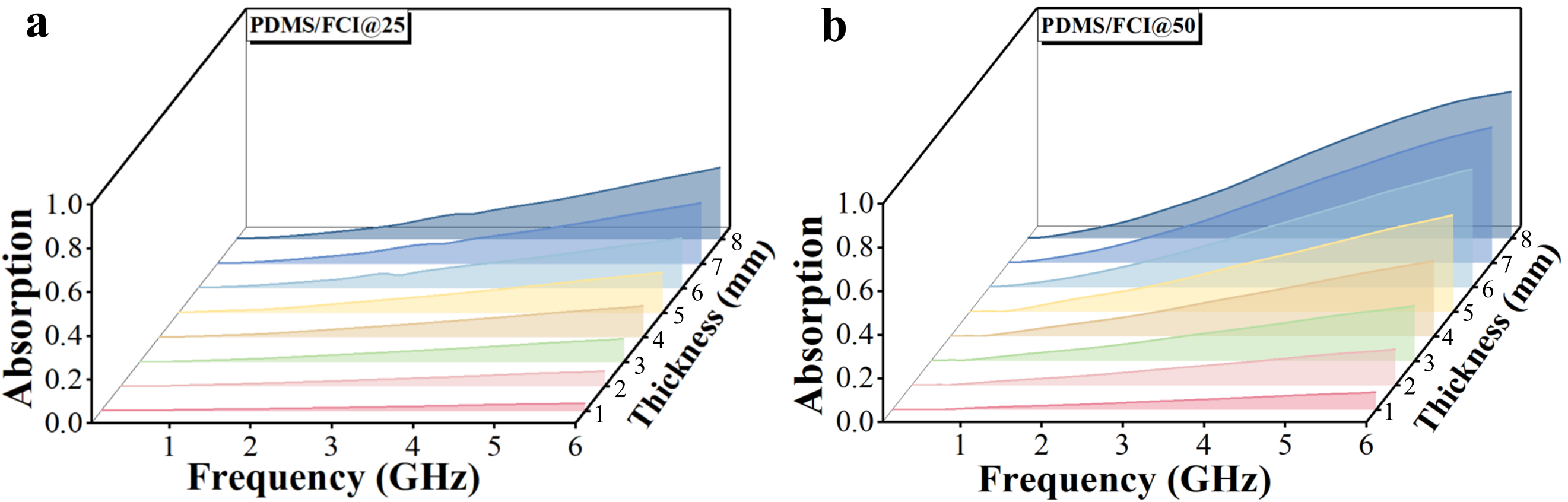
**

**Fig. S7** Absorption coefficient at thicknesses of 1-8 mm: **a** PDMS/FCI@25 composite; **b** PDMS/FCI@50 composite.

**S5 Details of absorption characteristics of CMFAC designed based on PDMS/FCI composites**

**
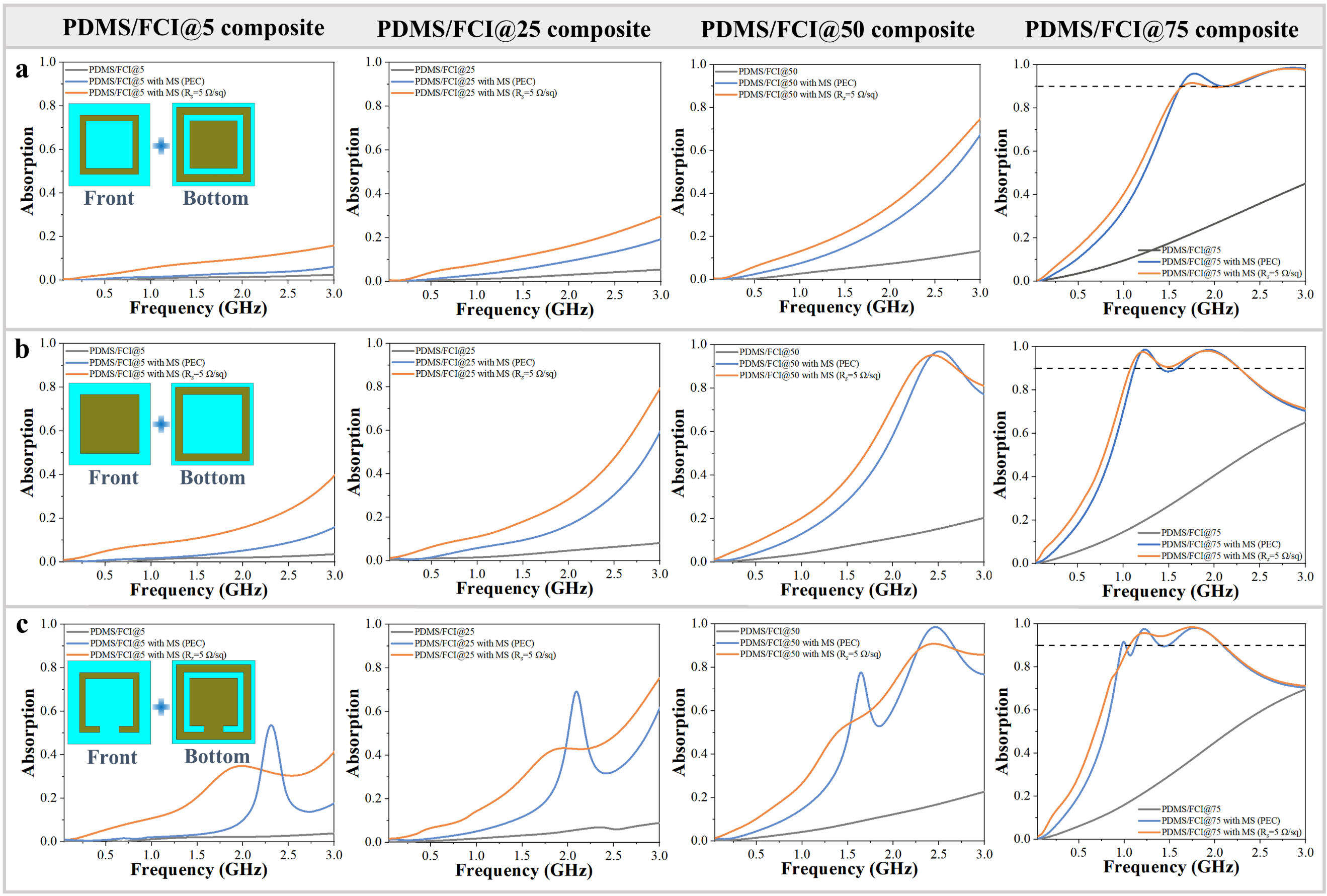
**

**Fig. S8** Comparison absorption coefficient of CMFACs under different parameter designs: **a** Square-ring MS, **b** Square MS, and **c** Square open ring MS.

**S6 Details of the angle-insensitive characteristics of the designed CMFAC**


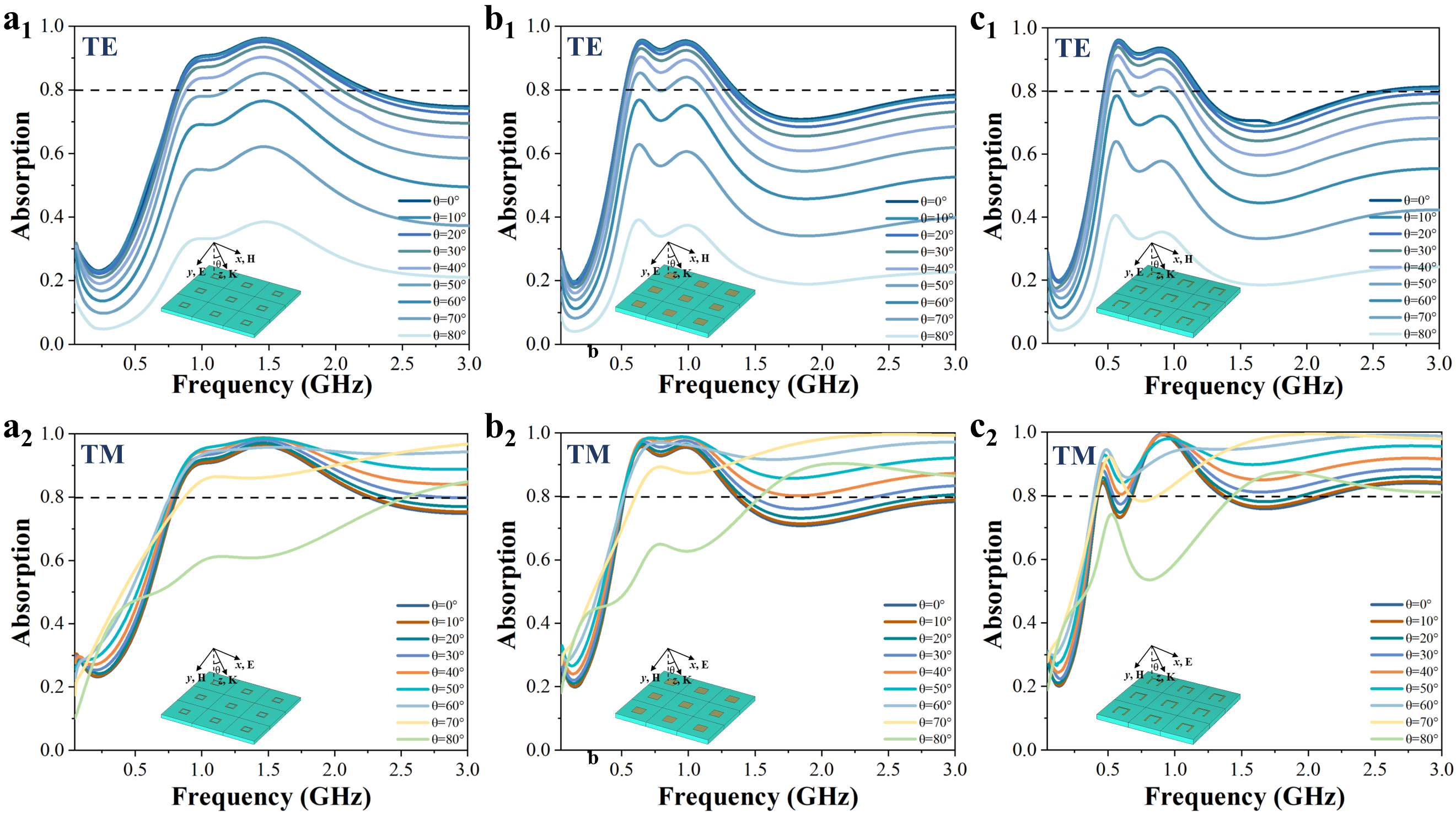


**Fig. S9** Simulated absorption coefficient of the CMFAC unit cell with different incident angles under TE and TM polarization: **a** Square-ring MS, **b** Square MS, and **c** Square open ring MS.

**S7 Details of the field distribution of the CMFAC**

**
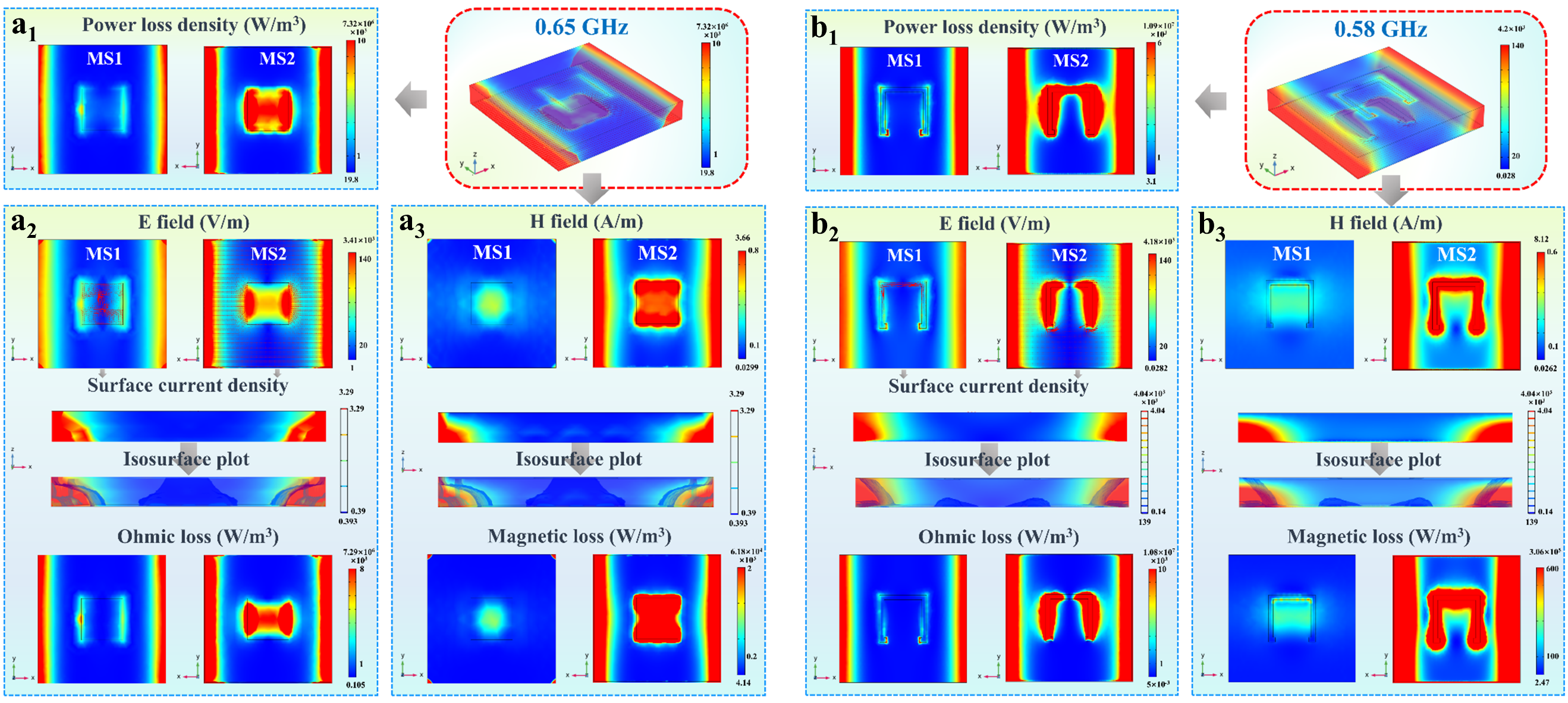
**

**Fig. S10** Simulated power loss density, electric field, magnetic field, and their corresponding isosurface plots, surface current, and loss distributions of the designed CMFACs under TE polarization: **a** Square MS, **b** Square open ring MS.

**S8 Details of the RCS of the CMFAC designed at phi=90°**


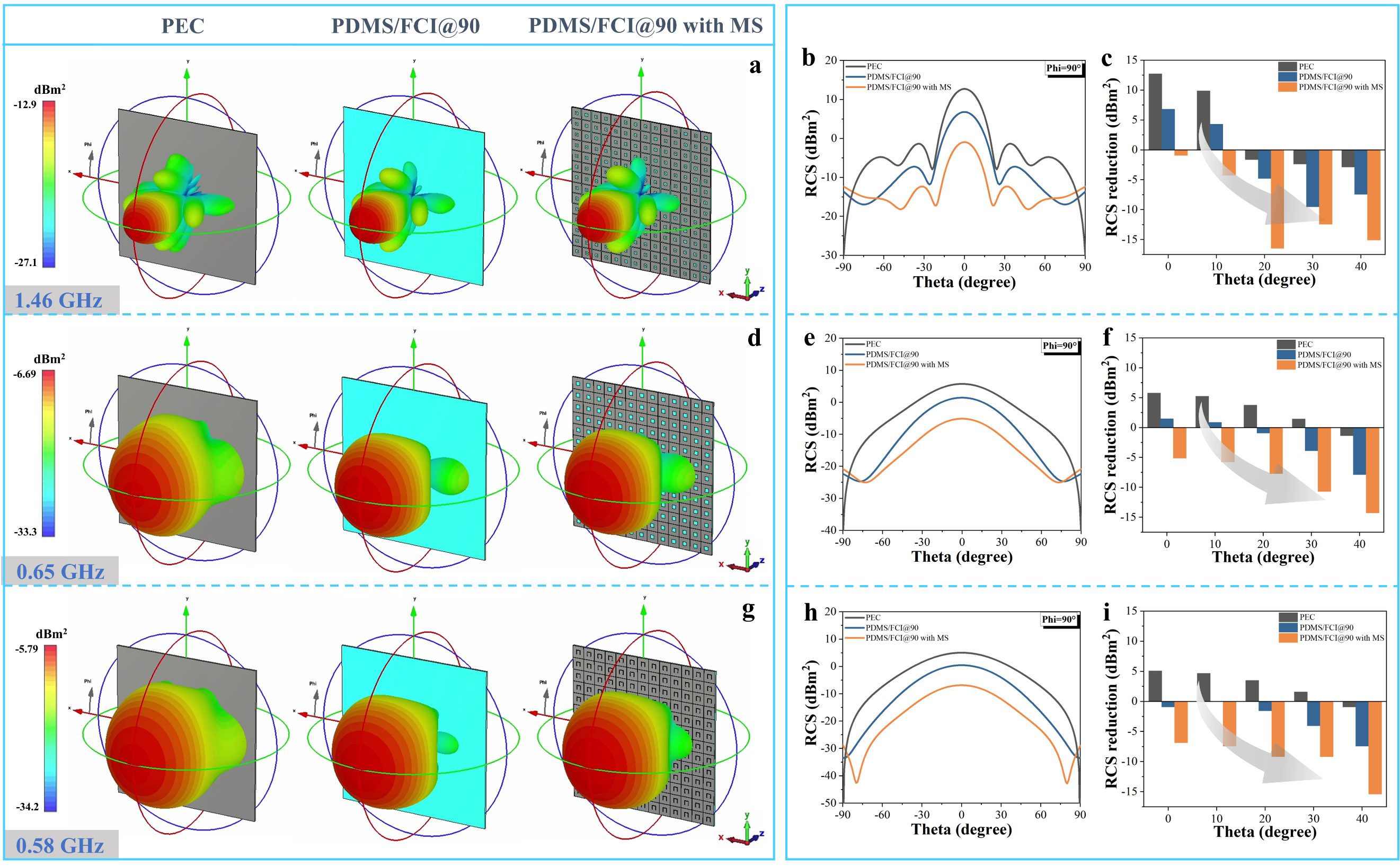


**Fig. S11** CMFAC with square-ring MS: **a** 3D far-field diagrams, **b** RCS plot in the cartesian coordinate system under certain detecting angles, and **c** RCS reduction values; CMFAC with square MS: **d** 3D far-field diagrams, **e** RCS plot in the cartesian coordinate system under certain detecting angles, and **f** RCS reduction values; CMFAC with square open ring MS: **g** 3D far-field diagrams, **h** RCS plot in the cartesian coordinate system under certain detecting angles, and **i** RCS reduction values.

**S9 Details for sample preparation**

**
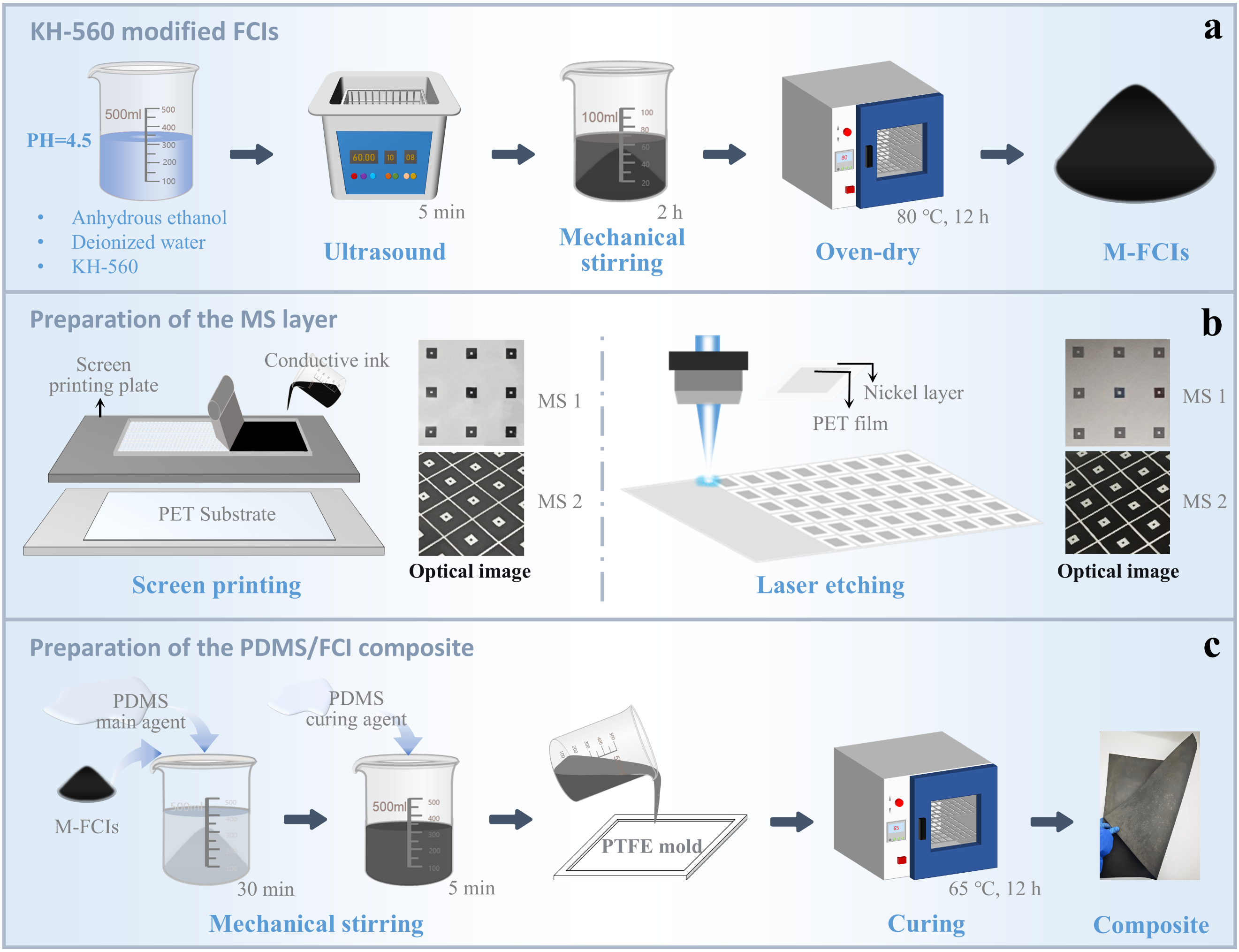
**

**Fig. S12** Schematic diagram of the CMFAC preparation process: **a** KH-560 modified FCIs; **b** MS layer; **c** PDMS/FCI composite.

**S10 Details of the components in the prepared CMFAC**


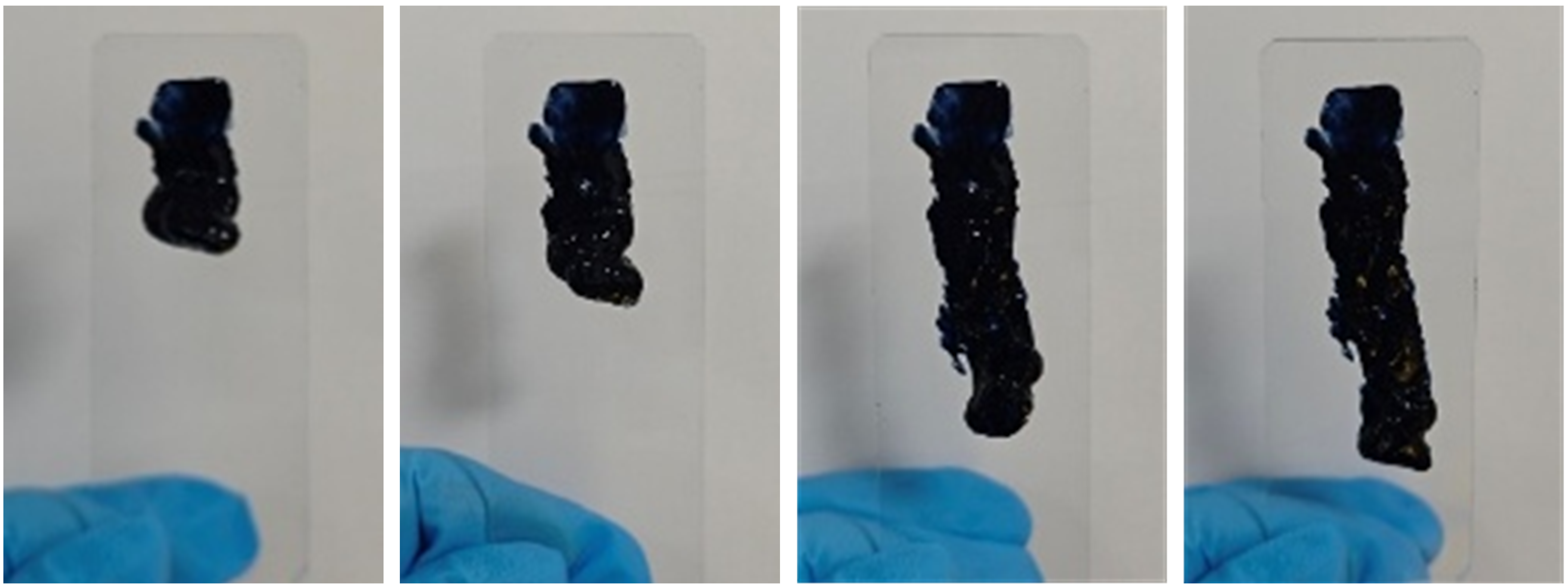


**Fig. S13** Actual image of flow behavior of 9% solids PEDOT: PSS conductive ink on a microscope slide.


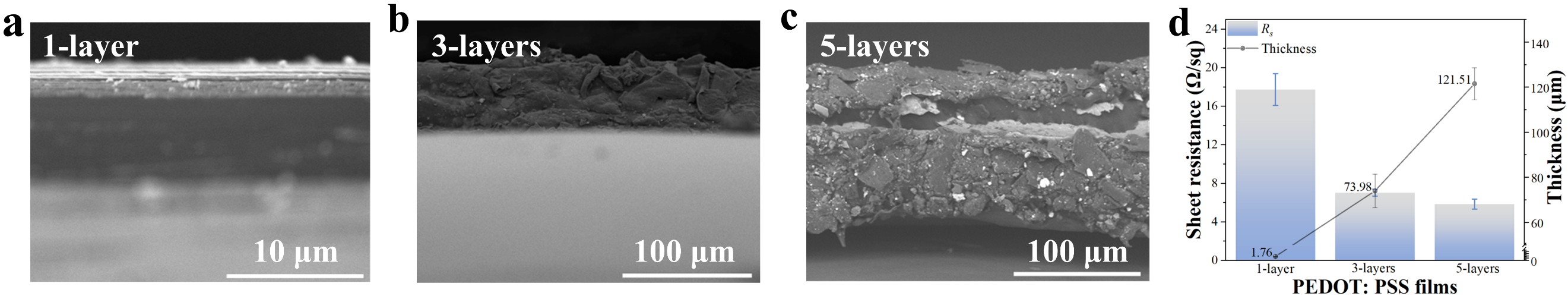


**Fig. S14** SEM image of the side view of PEDOT: PSS film after different numbers of screen-printing layers: **a** 1-layer; **b** 3-layers; **c** 5-layers; **d** Change in PEDOT: PSS film *R_s_* and thickness with number of printed layers.

**S11 Details for PDMS/FCI composites and Characterization**

***Mechanical Performance Test:*** Tensile performance test standard GB 528-1998, loading rate of 5 mm/min, continuous loading until the destruction of the specimen, record the maximum load value of the destruction of the specimen. Sample dimensions are as shown in the Fig S15a. Compression performance test standard GB 7759-1996, loading rate of 10 mm/min, continuous loading until the destruction of the specimen, record the maximum load value of the destruction of the specimen, until the predetermined strain is reached. For cyclic compression testing, the specimen undergoes repeated loading-unloading cycles at the same strain rate to evaluate its elastic recovery and energy dissipation capacity. Sample dimensions are as shown in the Fig S15b. Each group of specimens to test 5 valid samples, and record the average value.

**
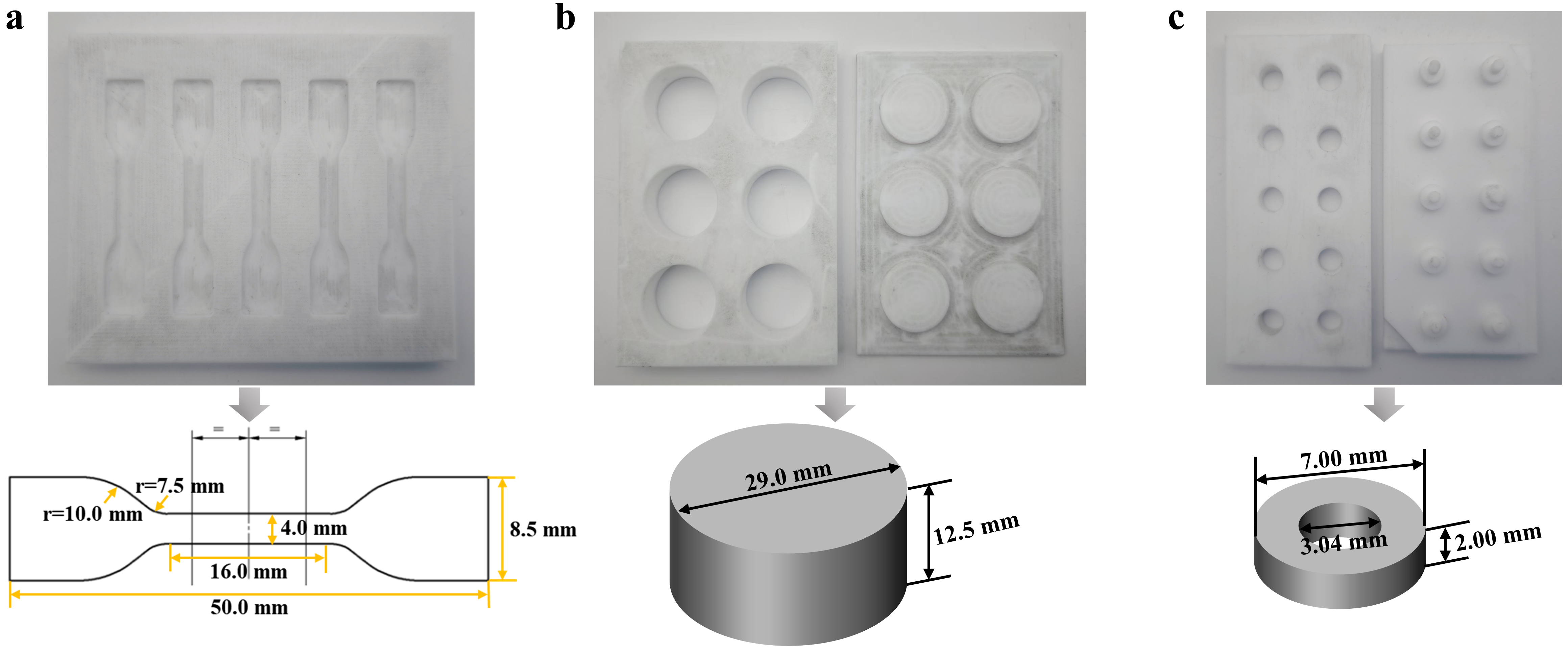
**

**Fig. S15** Preparation of PTFE molds for fabricating the PDMS/FCI composites of various specifications and their specific dimensions: **a** Tensile; **b** Compression; **c** Coaxial.


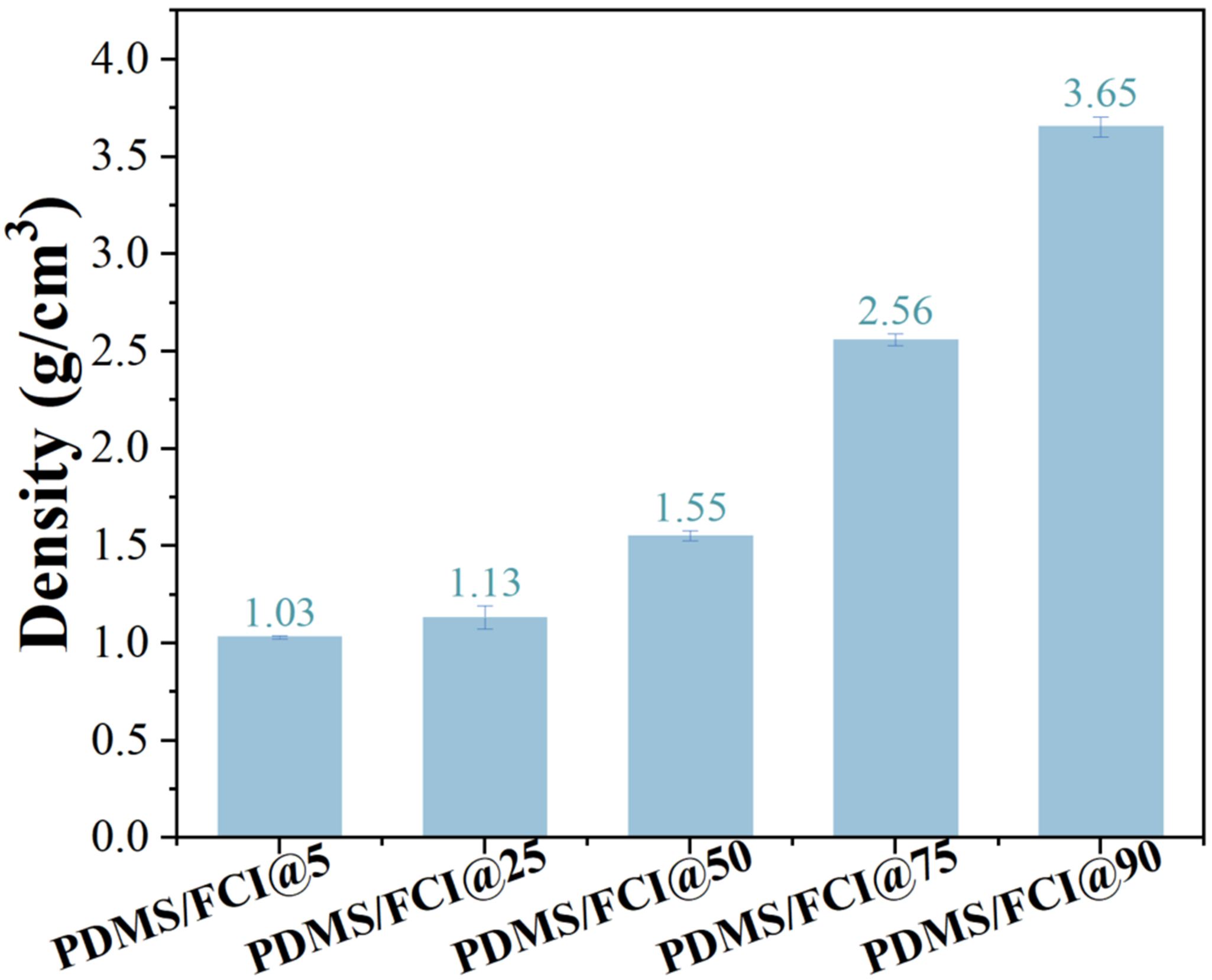


**Fig. S16** Density of the PDMS/FCI composites as a function of M-FCIs mass ratio.

**S12 Details for CST simulation**

**
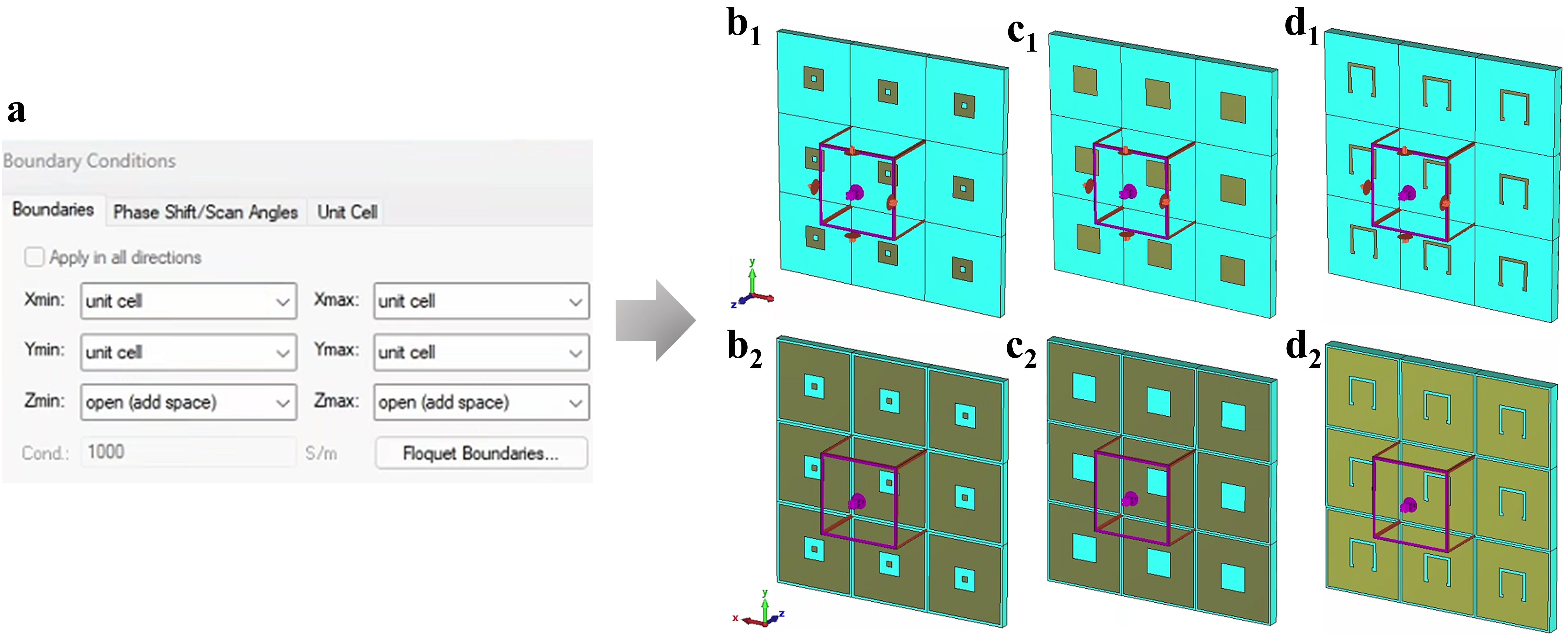
**

**Fig. S17 a** Boundary conditions for time-domain simulation; Frequency domain conditions with periodic boundaries: **b** Square-ring MS, **c** Square MS, and **d** Square open ring MS.

**Table S1** Simulation port

| **Simulation requirement** | **Excitation port** |
| --- | --- |
| Absorption coefficient of CMFAC units | Floquet port |
| Absorption coefficient of full-structure CMFAC | Wave port |
| Far field and near field of full-structure CMFAC | Plane wave port |

**Table S2** Optimization of CMFAC structural geometric parameters

| **Frequency Domain Solver, Genetic Algorithm** | | | | | | | |
| --- | --- | --- | --- | --- | --- | --- | --- |
| **MS** | **Settings** | | | | **Goals** | | |
|  | **Parameter** | **Min** | **Max** | **Best** | **Type** | **Target** | **Range** |
| Square- ring | P | 8.0 | 60.0 | 35.01 | Absorption coefficient | >0.9 | 0.3-3.0 |
|  | a (L_1_=P*a) | 0.1 | 1.0 | 0.94 |  |  |  |
|  | b (L_2_=P*a*b) | 0.1 | 1.0 | 0.28 |  |  |  |
|  | c (L_2_=P*a*b*c) | 0.1 | 1.0 | 0.25 |  |  |  |
|  | h | 1.0 | 8.0 | 3.78 |  |  |  |
| Square | P | 8.0 | 60.0 | 39.69 |  |  |  |
|  | a (L_1_=P*a) | 0.1 | 1.0 | 0.98 |  |  |  |
|  | b (L_2_=P*a*b) | 0.1 | 1.0 | 0.33 |  |  |  |
|  | h | 1.0 | 8.0 | 5.59 |  |  |  |
| Square open ring | P | 8.0 | 60.0 | 39.89 |  |  |  |
|  | a (L_1_=P*a) | 0.1 | 1.0 | 0.98 |  |  |  |
|  | b (L_2_=P*a*b) | 0.1 | 1.0 | 0.79 |  |  |  |
|  | c (L_3_=P*a*b*c) | 0.1 | 1.0 | 0.80 |  |  |  |
|  | d (L_4_=P*a*b*c*d) | 0.1 | 1.0 | 0.88 |  |  |  |
|  | h | 1.0 | 8.0 | 6.16 |  |  |  |

**Table S3** Genetic Algorithm Settings

| **Category** | **Parameter** | **Value** |
| --- | --- | --- |
| Generation settings | Population size | 8 |
|  | Max. number of iterations | 30 |
|  | Max. number of solver evaluations | 497 |
| Choice of initial point set | Latin Hypercube distribution | |
| General settings | Goal Function Level | 0 |
|  | Mutation rate | 60% |
|  | Random seed | 1 |

**S13** **Details for COMSOL simulation**

**Table S4** Simulation conditions

| **Simulation requirement** | **Excitation port** |
| --- | --- |
| Solver | Electromagnetic waves, frequency domain |
| Excitation source | Two driving sources, the input end and the receiving end |
| Boundary conditions | Periodic boundary condition |
|  | Scattering boundary condition |
|  | Perfectly matched layer |
| Grid division | Physical field control grid |

**S14 Details for CMFAC structural parameters**

To achieve the optimal absorption coefficient, we employed the genetic algorithm integrated in CST Microwave Studio to design and optimize the unit cell structure of the CMFAC. These parameters include the magnetic materials thickness *h*, the periodic unit cell *P*, the MS structure size *L*, and the MS layer material properties (PEC or *R_s_*). The fitness function of the algorithm was set to maximize the absorption coefficient within the target frequency band. The three CMFAC structures ultimately designed converged to a set of globally optimal structural parameters via the genetic algorithm. Detailed parameter specifications are provided in Table S4.

**Table S5** Dimensions of the CMFAC

|  | **Parameter (mm)** | | | | | | **Pattern Material Properties** | |
| --- | --- | --- | --- | --- | --- | --- | --- | --- |
|  | *h* | *P* | *L_1_* | *L_2_* | *L_3_* | *L_4_* |  |  |
| Square-ring | 3.78 | 35.0 | 33.1 | 9.3 | 2.3 | - | PEC | *R_s_=*5 Ω/sq |
| Square | 5.59 | 39.7 | 39.1 | 12.8 | - | - |  |  |
| Square open ring | 6.16 | 39.9 | 39.3 | 14.7 | 11.9 | 10.4 |  |  |

**S15 Details for LF absorbers**

**Table S6** Absorption performance comparison between the proposed CMFAC and 14 representative LF absorbers

|  | **Materials** | **Thickness (mm)** | **Equivalent thickness** | ***f*_min_**  **(GHz)** | **EAB**  **(GHz)** | **Ref.** |
| --- | --- | --- | --- | --- | --- | --- |
| **Ferrite matrix materials** | Ni_0.5_Zn_0.5_Nd_0.04_Fe_1.96_O_4_ | 8.50 | 0.0935λ | 3.30 | 3.30-6.30 | [S1] |
|  | Mn-Zn | 5.50 | 0.0477λ | 2.60 | 2.60-3.60 | [S2] |
| **Magnetic materials** | Co/CoO/SrCO_3_@C | 6.50 | 0.0331λ | 1.53 | 1.53-3.00 | [S3] |
|  | CoNi/Polydimethylsiloxane | 3.90 | 0.0343λ | 2.64 | 2.64-3.68 | [S4] |
| **Ceramic-based materials** | CMF/FeNi-SiO_2_ | 4.10 | 0.0400λ | 2.93 | 2.93-3.86 | [S5] |
|  | SiC_f_/FeNi/C | 5.00 | 0.0533λ | 3.20 | 3.20-3.67 | [S6] |
| **Conductive polymer materials** | [Fe_3_O_4_@PANI](mailto:Fe3O4@PANI) | 4.85 | 0.0425λ | 2.63 | 2.63-3.57 | [S7] |
|  | PC/Fe_3_O_4_@PDA | 4.03 | 0.0578λ | 4.30 | 4.30-7.81 | [S8] |
| **Carbon-based materials** | Co_3_O_4_-MWCNT | 7.22 | 0.0657λ | 2.73 | 2.73-4.22 | [S9] |
|  | Co/rGO | 6.40 | 0.0655λ | 3.07 | 3.07-4.06 | [S10] |
| **MXene-based materials** | [MXene@gelatin](mailto:MXene@gelatin) | 4.37 | 0.0538λ | 3.69 | 3.69-4.64 | [S11] |
|  | [CoNiZn@Ti_3_CNT_x_@CF](mailto:CoNiZn@Ti3CNTx@CF) | 5.50 | 0.0631λ | 3.44 | 3.44-4.30 | [S12] |
| **Metamaterials** | Ferrite + MM | 4.13 | 0.0551λ | 4.00 | 4.00-10.0 | [S13] |
|  | CIP + MM | 4.00 | 0.0293λ | 2.20 | 2.20-8.00 | [S14] |
|  | **PDMS/FCI@90 + MMs** | **3.78** | **0.0120λ** | **0.95** | **0.95-1.81** | **This work** |
|  |  | **5.59** | **0.0106λ** | **0.57** | **0.57-1.16** |  |
|  |  | **6.16** | **0.0105λ** | **0.51** | **0.51-1.03** |  |
|  | **PDMS/FCI@75 + MMs** | **3.78** | **0.0223λ** | **1.77** | **1.77-2.85** |  |

**Supplementary References**

[S1] K. Qian, Z. Yao, H. Lin, J. Zhou, A. A. Haidry et al., The influence of Nd substitution in Ni-Zn ferrites for the improved microwave absorption properties. Ceram. Int. **46**, 227-235 (2020). https://doi.org/10.1016/j.ceramint.2019.08.255.

[S2] Y. Zhang, Z. Kang, D. Chen, Synthesis and microwave absorbing properties of Mn-Zn nanoferrite produced by microwave assisted ball milling. J Mater Sci: Mater Electron. **25**, 4246-4251 (2014). <https://doi.org/10.1007/s10854-014-2156-z>.

[S3] L. Zhang, P. Yin, J. Wang, X. Feng, J. Dai, Low-frequency microwave absorption of MOF-derived Co/CoO/SrCO_3_@C composites. Mater. Chem. Phys. **264**, 124457 (2021). <https://doi.org/10.1016/j.matchemphys.2021.124457>.

[S4] M. He, J. Hu, H. Yan, X. Zhong, Y. Zhang et al., Shape anisotropic chain-like CoNi/Polydimethylsiloxane composite films with excellent low-frequency microwave absorption and high thermal conductivity. Adv. Funct. Mater. **35**, 2316691 (2025). <https://doi.org/10.1002/adfm.202316691>.

[S5] Y. Zou, X. Huang, B. Fan, J. Yue, Y. Liu, Enhanced low-frequency microwave absorption performance of FeNi alloy coated carbon foam assisted by SiO_2_ layer. Appl. Surf. Sci. **600**, 154046 (2022). <https://doi.org/10.1016/j.apsusc.2022.154046>.

[S6] T. Guo, B. Huang, C. Li, Y. Lou, X.-Z. Tang et al., Magnetic sputtering of FeNi/C bilayer film on SiC fibers for effective microwave absorption in the low-frequency region. Ceram. Int. **47**, 5221-5226 (2021). <https://doi.org/10.1016/j.ceramint.2020.10.101>.

[S7] X. Luo, H. Li, D. Deng, L. Zheng, Y. Wu et al., Preparation and excellent electromagnetic absorption properties of dendritic structured Fe_3_O_4_@PANI composites. J. Alloys Compd. **891**, 161922 (2022). https://doi.org/10.1016/j.jallcom.2021.161922.

[S8] Y. Xie, Y. Guo, T. Cheng, L. Zhao, T. Wang et al., Efficient electromagnetic wave absorption performances dominated by exchanged resonance of lightweight PC/Fe_3_O_4_@PDA hybrid nanocomposite. Chem. Eng. J. **457**, 141205 (2023). <https://doi.org/10.1016/j.cej.2022.141205>.

[S9] J.-C. Shu, X.-Y. Huang, M.-S. Cao, Assembling 3D flower-like Co_3_O_4_-MWCNT architecture for optimizing low-frequency microwave absorption. Carbon N. Y. **174**, 638-646 (2021). <https://doi.org/10.1016/j.carbon.2020.11.087>.

[S10] M. Fu, Q. Jiao, Y. Zhao, H. Li, Vapor diffusion synthesis of CoFe_2_O_4_ hollow sphere/graphene composites as absorbing materials. J Mater Chem A. **2**, 735-744 (2014). https://doi.org/10.1039/c3ta14050d.

[S11] M. Yang, Y. Yuan, Y. Li, X. Sun, S. Wang et al., Anisotropic electromagnetic absorption of aligned Ti_3_C_2_T_x_ MXene/gelatin nanocomposite aerogels. ACS Appl. Mater. Interfaces. **12**, 33128-33138 (2020). <https://doi.org/10.1021/acsami.0c09726>.

[S1**2**] R. Tan, W. Bai, J. Yan, L. Geng, S. Jiang et al., Flexible CoNiZn@Ti_3_CNT_x_ MXene@carbon fabrics with hierarchical structure for efficient electromagnetic wave absorption. Chem. Eng. J. **500**, 157444 (2024). <https://doi.org/10.1016/j.cej.2024.157444>.

[S13] Z. Yang, Y. Che, X. Sun, J. Zhang, J. Tian et al., Broadband polarization-insensitive microwave-absorbing composite material based on carbon nanotube film metamaterial and ferrite. J. Appl. Phys. **125**, 13838-13844 (2020). <https://doi.org/10.1063/1.5086315>.

[S14] Z. Zhang, L. Zhang, X. Chen, Z. Wu, Y. He et al., Broadband metamaterial absorber for low-frequency microwave absorption in the S-band and C-band. J. Magn. Magn. Mater. **497**, 166075 (2020). <https://doi.org/10.1016/j.jmmm.2019.166075>.
